# Does the Red Shift in UV–Vis Spectra Really Provide a Sensing Option for Detection of *N*-Nitrosamines Using Metalloporphyrins?

**DOI:** 10.1021/acsomega.2c06615

**Published:** 2022-12-20

**Authors:** Marko Trampuž, Mateja Žnidarič, Fabrice Gallou, Zdenko Časar

**Affiliations:** † Lek Pharmaceuticals d.d., Sandoz Development Center Slovenia, Kolodvorska 27, 1234 Mengeš, Slovenia; ‡ Chemical and Analytical Development, Novartis Pharma AG, Basel 4056, Switzerland; § Chair of Medicinal Chemistry, Faculty of Pharmacy, University of Ljubljana, Aškerčeva cesta 7, 1000 Ljubljana, Slovenia

## Abstract

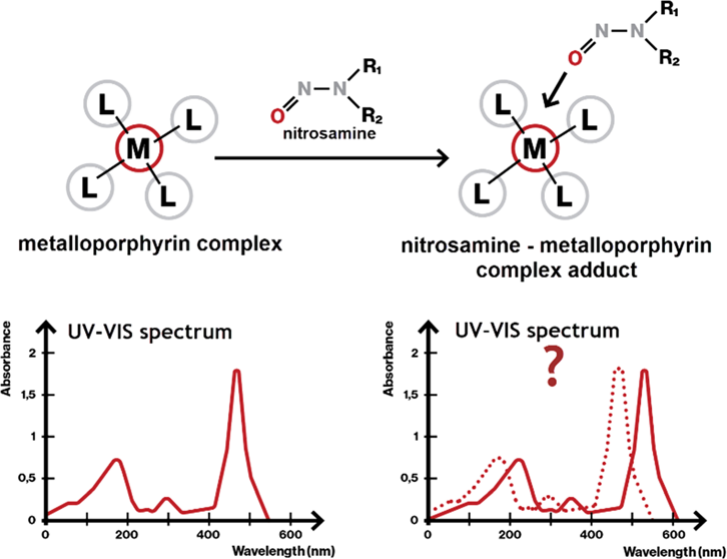

*N*-nitrosamines are widespread cancerogenic compounds in human environment, including water, tobacco products, food, and medicinal products. Their presence in pharmaceuticals has recently led to several recalls of important medicines from the market, and strict controls and tight limits of *N*-nitrosamines are now required. Analytical determination of *N*-nitrosamines is expensive, laborious, and time-inefficient making development of simpler and faster techniques for their detection crucial. Several reports published in the previous decade have demonstrated that cobalt porphyrin-based chemosensors selectively bind *N*-nitrosamines, which produces a red shift of characteristic Soret band in UV–Vis spectra. In this study, a thorough re-evaluation of metalloporphyrin/*N*-nitrosamine adducts was performed using various characterization methods. Herein, we demonstrate that while *N*-nitrosamines can interact directly with cobalt-based porphyrin complexes, the red shift in UV–Vis spectra is not selectively assured and might also result from the interaction between impurities in *N*-nitrosamines and porphyrin skeleton or interaction of other functional groups within the *N*-nitrosamine structure and the metal ion within the porphyrin. We show that pyridine nitrogen is the interacting atom in tobacco-specific *N*-nitrosamines (TSNAs), as pyridine itself is an active ligand and not the *N*-nitrosamine moiety. When using Co(II) porphyrins as chemosensors, acidic and basic impurities in dialkyl *N*-nitrosamines (e.g., formic acid, dimethylamine) are also UV–Vis spectra red shift-producing species. Treatment of these *N*-nitrosamines with K_2_CO_3_ prevents the observed UV–Vis phenomena. These results imply that cobalt-based metalloporphyrins cannot be considered as selective chemosensors for UV–Vis detection of *N*-nitrosamine moiety-containing species. Therefore, special caution in interpretation of UV–Vis red shift for chemical sensors is suggested.

## 1 Introduction

**Figure 1:**
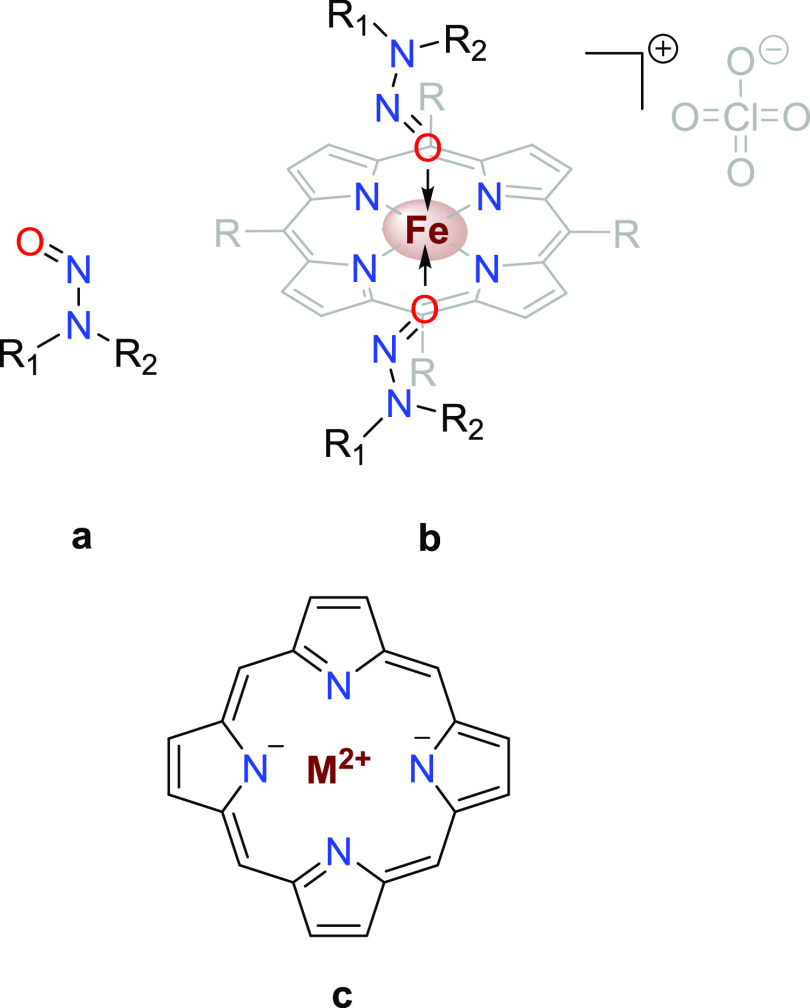
(a) General structure of *N*-nitrosamines, (b) structure of *N*-nitrosamine/metalloporphyrin complex [(TPP)Fe(ONNR_2_)_2_]ClO_4_, and (c) general structure of metalloporphyrins.

*N*-Nitrosamines are a class of cancerogenic compounds, which contain a nitroso functional group directly bound to a nitrogen atom (Figure 1a).^1^ Their biological mechanism of carcinogenicity is metabolic activation by cytochrome P450 enzymes to highly reactive diazonium species, which may alkylate DNA, leading to mutations and oncogenesis.^2−6^ They are ubiquitous in human environment, as they may be found in various amounts in air, soil, water, and various products that humans use daily, such as food,^7^ medicinal products,^8^ cosmetics,^9^ rubber,^10^ etc. Their presence in cigarette smoke and drinking water is also widely acknowledged.^11−14^ In recent years, several medicinal products have been found to be contaminated with unacceptably high levels of various *N*-nitrosamines. As a result, several medicines had to be withdrawn from the market. Consequently, regulatory agencies, including Food and Drug Administration (FDA) and European Medicines Agency (EMA), imposed strict control of medicinal products regarding the presence of *N*-nitrosamines.^8,15^ They are also included in the cohort of concern in the ICH M7 guidelines as classes of mutagens with an extremely high carcinogenic potency.^16,17^

The current *N*-nitrosamine risk assessment approach^18−20^ in the pharmaceutical industry consists of paper-based evaluation of synthetic routes and subsequent GC-MS and/or LC-MS analysis^21−27^ of drug substances and drug products considered to be under risk for *N*-nitrosamine contamination. The current instrumental analytical methods that can quantify *N*-nitrosamines in levels below ppm region are time-consuming and expensive, require highly skilled personnel, and require development of the specific analytical method for each individual *N*-nitrosamine and each drug product. However, there could be a wide plethora of structurally different *N*-nitrosamines present in drug products, with nitrosamine drug substance-related impurities (NDSRI) posing a particular concern.^28^ Development of faster, simpler tests for simultaneous cumulative detection of various *N*-nitrosamines at sufficiently low levels (ppb range) within diverse pharmaceutical samples with simple analytical methods is therefore of utmost importance. Various novel analytical techniques have been developed recently to detect *N*-nitrosamines in samples of various complexity, such as two square-wave voltammetry using a boron-doped diamond electrode for vegetable samples,^29^ supramolecular fluorescent sensors based on two cucurbit[*n*]uril probes,^30^ impedimetric sensors based on molecularly imprinted polymers,^31^ UV photolysis with chemiluminescence detection of released nitric oxide,^32^ and differential pulse voltammetry using DNA carbon-nanodot-modified electrodes^33^ for aqueous samples, and carbon nanotube-based chemiresistive sensors, utilizing cobalt(III) tetraphenylporphyrin^34^ and metallocalix[4]arene polymers with gravimetric detection^35^ for air samples. However, to the best of our knowledge, recent reports have presented only one novel method for *N*-nitrosamine detection in medicines, which is reduction to electrochemically active hydrazines and quantitative determination of the latter with coulometric mass spectrometry without using standards.^36^

The interaction of *N*-nitrosamines with cytochrome P450 enzymes has been known since 1979, where two types of interactions were presented: type I (interaction with the protein part) and type II (interaction with the iron center in heme).^37^ In 1995, Richter-Addo et al. presented the structure of an isolated octahedral complex between (*meso*-tetraphenylporphyrinato)iron(III) perchlorate and *N*-nitrosodiethylamine (NDEA), where two molecules of the *N*-nitrosamine bind axially with the oxygen atom to the iron metal of the metalloporphyrin (Figure 1b).^38^ Several other metalloporphyrins (general structure shown in Figure 1c), such as those based on ruthenium and osmium, were later found in a larger study by the same research group to form such complexes.^39^ Another follow-up study demonstrated that both aliphatic and aromatic *N*-nitrosamines bind in this way.^40^ These studies affirmed the *O*-binding mode of the *N*-nitrosamine group to metal centers in metalloporphyrins and revealed the ability of *N*-nitrosamines to function as *O*-donor ligands.^38−41^

Interestingly, a study by Dai et al. showed that a distinct change in the UV–Vis spectrum of a cobalt metalloporphyrin occurs upon binding of tobacco-specific *N*-nitrosamines (TSNAs) in solution, where the characteristic Soret band of the studied metalloporphyrin at 414 nm disappeared upon increasing the concentration of TSNAs and a novel peak at 438 nm appeared.^13^ Furthermore, such a bathochromic shift, also known as a red shift, was also shown to occur with a different cobalt metalloporphyrin upon addition of TSNAs, which was later attributed to the ability of the −N–NO functional group in the TSNAs to bond with the cobalt center of the porphyrins.^42^ Formation of *N*-nitrosamine/metalloporphyrin complexes stipulated the use of metalloporphyrins as UV–Vis chemosensors for the presence of *N*-nitrosamines, and the previously mentioned study by Swager et al. demonstrated the applicability of (*meso*-tetraphenylporphyrinato)cobalt(III) perchlorate adsorbed on carbon nanotubes as a chemiresistive sensor to selectively detect volatile dialkyl *N*-nitrosamines in air in low ppb levels.^34^

The significant change that occurs in the metalloporphyrin UV–Vis spectra upon binding of *N*-nitrosamines to metalloporphyrins in recent reports^13,34,42^ therefore encouraged us to pursue the development of selective UV–Vis chemosensors for detection of *N*-nitrosamines in medicinal products. After initial evaluation of the already known metalloporphyrins that interact with *N*-nitrosamines, the aim of our study was to find the right metal ion for the chemosensor and then optimize the porphyrin chemical structure to achieve the desired selectivity and sensitivity. A wide variety of metal ions may be complexed by porphyrins and porphyrin composites, including palladium and platinum, and such complexes might also exhibit chemosensing properties for *N*-nitrosamines.^43^ Furthermore, larger rings, such as benzoporphyrins, may offer advantageous optical and photophysical properties.^44^ However, a careful analysis of the presented metalloporphyrins and their exposure to various *N*-nitrosamines under different conditions revealed that the observed red shift in the UV–Vis spectrum of metalloporphyrins may be influenced by a wide variety of different compounds present as impurities in *N*-nitrosamines. The aim of this paper is therefore to present the results of our detailed investigation in the changes in UV–Vis spectra of metalloporphyrins upon exposure to *N*-nitrosamines and various other chemical species, which also explains many of the results presented in the recent reports on *N*-nitrosamine/metalloporphyrin interaction and gives guidelines for future research in the field of *N*-nitrosamine analytics and development of metalloporphyrin-based chemosensors in general.

## 2 Results and Discussion

### 2.1 Evaluation of the Previous Reports on *N*-Nitrosamine Chemosensors Based on Metalloporphyrins

Three recent literature reports triggered our attention to metalloporphyrins as potential UV–Vis chemosensors for *N*-nitrosamines in medicinal products.^13,34,42^ A detailed reading of these reports revealed some inconsistencies in reported data. In this section, we would therefore like to list these and present the results of our reproduction of the performance of these metalloporphyrins as *N*-nitrosamine chemosensors.

#### 2.1.1 Evaluation of the Porphyrin F Interaction with Tobacco-Specific *N*-Nitrosamines

**Figure 2:**
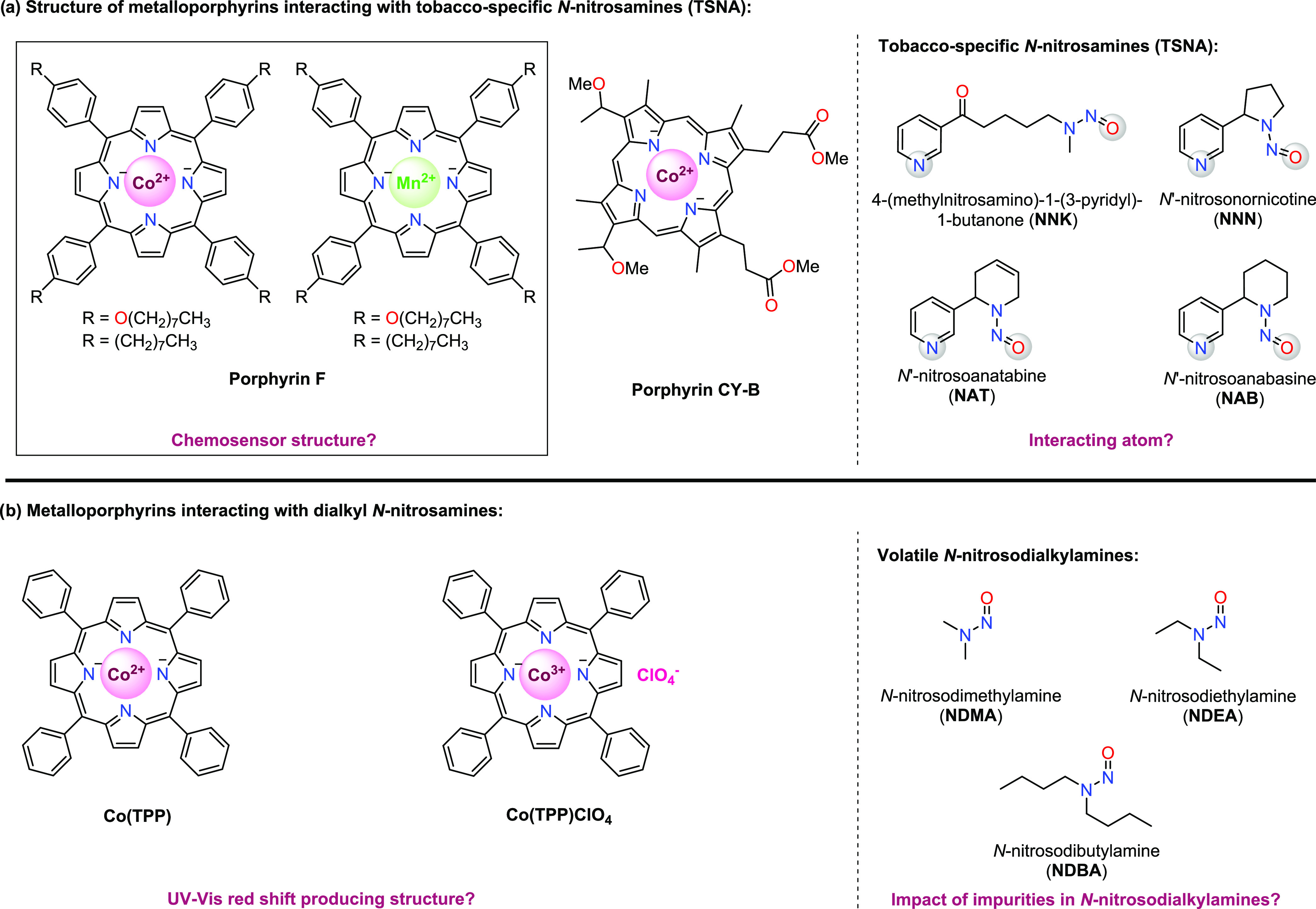
Structures of the reported metalloporphyrin-based chemosensors: (a) porphyrin F (four possible structures) and metalloporphyrin CY-B for TSNAs and (b) Co(TPP) and Co(TPP)ClO_4_ for dialkyl *N*-nitrosamines.

The paper by Dai et al. presents two different structures of the so-called porphyrin F: *meso*-tetra-(4-octoxylphenyl) manganese porphyrin and *meso*-tetra-(4-octylphenyl) cobalt porphyrin, which differ in the metal atom present in the porphyrin core and the nature of the side chain on the four phenyl substituents.^13^ However, we also could not exclude that the other two combinations of metals and porphyrin ligands presented in the report of Dai et al.^13^ were the correct ones, so porphyrin F could be any of the four possible structures, as shown in Figure 2a. To the best of our knowledge, only the interaction between manganese porphyrins and nitrosoarenes was evaluated in the literature so far,^45^ while such an interaction has not been reported for *N*-nitrosamines yet. Due to these ambiguities, we decided to investigate the correct porphyrin F structure and thus synthesized all four possible compounds, according to the published literature procedures.^46^

Soret band of each molecule was compared to the results that were presented in the Dai’s report.^13^ Porphyrin F had a reported Soret band at 414 nm, and upon the reaction with TSNAs, the band was red-shifted to 438 nm. Two manganese porphyrins had a Soret band at 480 nm, indicating that porphyrin F does not contain a manganese ion. *Meso*-tetra-(4-octylphenyl) cobalt porphyrin had a Soret band at 412 nm, while the Soret band of *meso*-tetra-(4-octoxylphenyl) cobalt porphyrin was at 415 nm. This indicates that porphyrin F is one of the two cobalt porphyrins, but due to the close resemblance in the UV–Vis spectra, we could not identify whether the substituents of the porphyrin core are octyl or octyloxy chains. The measured UV–Vis spectra of all four compounds may be found in the Supporting Information (Figure S1). We repeated the titrations of both possible cobalt-containing porphyrins F with 1–50 equiv of the four studied TSNAs: *N*′-nitrosoanabasine (NAB), *N*′-nitrosoanatabine (NAT), 4-(methylnitrosamino)-1-(3-pyridyl-)-1-butanone (NNK), and *N*′-nitrosonornicotine (NNN). We were able to replicate the published UV–Vis spectra (Figures S2 and S3) with all four TSNAs. Under careful observation, one may see that the wavelengths of the red-shifted peaks differ a bit, depending on the metalloporphyrin and TSNA used. Since the red-shifted peaks at 432–438 nm appeared for *meso*-tetra-(4-octylphenyl) cobalt porphyrin [Co(OTPP)] and 437–441 nm for *meso*-tetra-(4-octoxylphenyl) cobalt porphyrin [Co(OOTPP)], we may conclude that the latter is the correct porphyrin F structure. This suggests that the used compound is none of the two structures described in the paper.

#### 2.1.2 Evaluation of the Metalloporphyrin CY-B Interaction with Tobacco-Specific *N*-Nitrosamines

The follow-up study by the same authors in 2014 presented a different porphyrin named CY-B (Figure 2a), a cobalt protoporphyrin IX derivative, which binds two molecules of TSNAs via the interaction between cobalt metal and the *N*-nitrosamine functional group.^42^ A red shift from 393 to 418 nm was observed in the measured UV–Vis spectra after TSNA addition to metalloporphyrin CY-B. We resynthesized metalloporphyrin CY-B and repeated the UV–Vis measurements, which were in accordance with the published ones (Figure S4). Dai and Yu et al. also confirmed the formation of complexes between metalloporphyrin CY-B and TSNAs by high-resolution mass spectrometry (HR-MS).^42^ Three mass peaks can be observed on the spectra, one of which belongs to the pure metalloporphyrin CY-B and two mass peaks that correspond to one and two additional TSNA molecules being coordinated to metalloporphyrin CY-B, respectively.^42^ Since we did not have HR-MS at our disposal, the binding of NNN as representative TSNA to metalloporphyrin CY-B was studied using ESI-MS. Interestingly, the authors claimed that the solutions used for UV–Vis spectroscopy were analyzed subsequently by HR-MS. This is a rather unusual approach due to CH_2_Cl_2_ used in UV–Vis measurement being too nonpolar (dielectric constant ε = 9.1) and thus less suitable as MS solvent due to weak stabilization of charged species.^47^ Our UV–Vis samples (with red-shifted metalloporphyrin CY-B Soret band due to NNN binding) were thus diluted 10 times with methanol prior to MS analysis. The formation of 1:1 and 1:2 CY-B/NNN complexes was indeed observed, as evidenced by peaks with *m*/*z* 711.0, 888.0, and 1065.2. Similar measurements were conducted with the other three TSNAs (Figure S5). However, the proposed interaction of TSNAs to cobalt metal caught our attention. Because pyridine-type nitrogen atoms are Lewis bases and therefore known ligands for metal porphyrins,^48−55^ the interaction between metalloporphyrin and TSNAs via pyridine nitrogen cannot be excluded. Interestingly, Tsutsui’s study in 1969 already showed that metalloporphyrin CY-B may coordinate pyridine, which changes its UV–Vis spectral and magnetic properties.^54^ Nevertheless, Dai and Yu et al.^42^ did not consider this possibility for TSNAs at all.

#### 2.1.3 Evaluation of the Co(TPP)ClO_4_ Interaction with *N*-Nitrosamines

**Figure 3:**
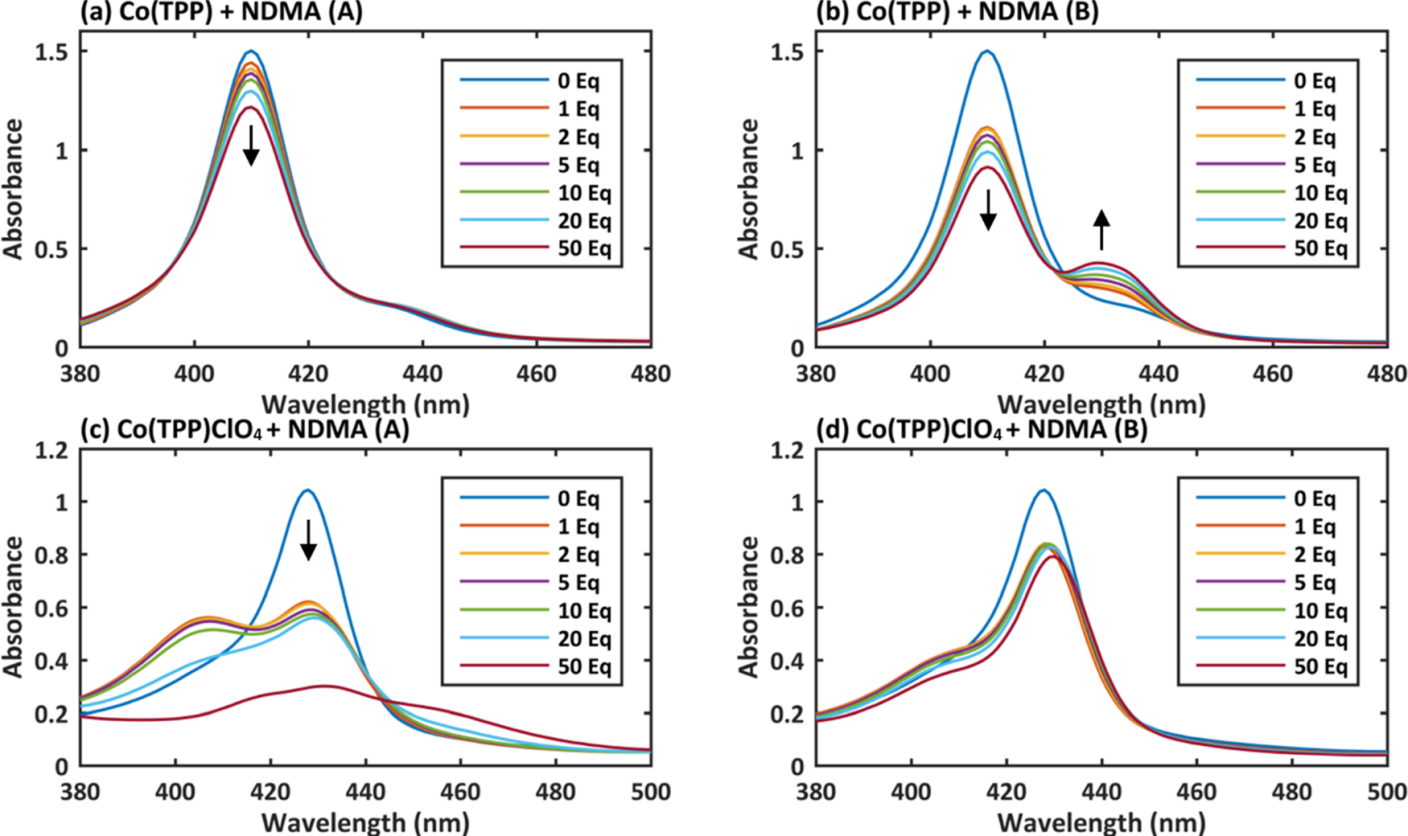
UV–Vis spectra of Co(TPP) titrated with (a) NDMA (supplier A), (b) NDMA (supplier B), and Co(TPP)ClO_4_ titrated with (c) NDMA (supplier A) and (d) NDMA (supplier B).

Interestingly, a cobalt metalloporphyrin bound to 4-pyridyl (7,6)-single-walled carbon nanotubes (SWCNTs) was utilized in the third study in question by Swager et al. to prepare the studied chemiresistive sensor for detection of *N*-nitrosamines in air.^34^ The study focused on volatile *N*-nitrosodialkylamines, such as *N*-nitrosodimethylamine (NDMA), NDEA, and *N*-nitrosodibutylamine (NDBA), and not on TSNAs, as in previously mentioned studies. However, some discrepancies may be observed in the paper, which have caught our attention. The correct structure of the studied chemosensor was dubious in this case (Figure 2b), as Co(TPP)ClO_4_ is mentioned throughout the manuscript text, while the presented red shift from 410 to 430 nm upon addition of NDMA (0–38 equiv) to Co(TPP)ClO_4_ is ascribed to Co(TPP). Moreover, the description of the presented red shift incorrectly states that the red shift from 310 to 330 nm was observed upon addition of NDMA.^34^ While the latter may likely be the result of a typing error, we wanted to be sure about the correct structure of the studied chemosensor. Co(TPP)ClO_4_ was synthesized according to the published procedure,^55^ and both Co(TPP) and Co(TPP)ClO_4_ were then separately titrated with NDMA and monitored by UV–Vis spectroscopy (Figure 3). The Soret band at 410 nm in the reported figure indeed corresponds to Co(TPP) solution in CH_2_Cl_2_. Co(TPP)ClO_4_ in CH_2_Cl_2_ exhibits its main absorption peak at 430 nm, which is also backed by literature information.^55^ While the authors stated that they used Co(TPP)ClO_4_ as the chosen chemosensor due to its highest response when bound to SWCNTs compared to other metal complexes of *meso*-tetraphenylporphyrin, there was no explanation provided why the observed red shift in UV–Vis study was attributed to Co(TPP). As a result of this inconsistency, one could question the valid identity of the metalloporphyrins in the UV–Vis experiments. Furthermore, Figure 3 shows that upon titration of both Co(TPP) and Co(TPP)ClO_4_ with NDMA, the results could only be replicated with certain batches of NDMA originating from different suppliers. Addition of NDMA provided by one supplier (A; see Section 4) did not lead to a red shift in the UV–Vis spectrum of Co(TPP)’s Soret band (Figure 3a), while NDMA provided by another supplier (B; see Section 4) produced a red shift at 434 nm with Co(TPP) (Figure 3b). Surprisingly, NDMA by supplier A caused a blue shift of Co(TPP)ClO_4_’s Soret band to 410 nm (Figure 3c), which coincides with Co(TPP), indicating a possible reduction. The UV–Vis spectrum became more complex upon addition of more than 20 equiv of NDMA. On the other hand, no change in the UV–Vis spectrum of Co(TPP)ClO_4_ upon addition of NDMA by supplier B was observed (Figure 3d). These observations led us to hypothesize that the quality of various *N*-nitrosamine batches may have an important effect on the possibility to detect *N*-nitrosamines using metalloporphyrin-based UV–Vis chemosensors. An investigation of their purity was thus commenced and will be discussed in subsequent sections.

Swager et al. carried out two more spectroscopic studies to confirm the interaction between Co(TPP)ClO_4_ and NDMA. The ^1^H NMR study of consequential addition of NDMA to Co(TPP)ClO_4_ showed a transition of the cobalt complex from a paramagnetic compound, showing no signals in NMR, to a diamagnetic state. The latter is explained by the authors to be the result of binding of NDMA to Co(TPP)ClO_4_, which creates a strong ligand field. There was however no information on the amount or stoichiometry of NDMA used for the titration, making the experiment impossible to repeat precisely. Our ^1^H NMR spectra of Co(TPP) and Co(TPP)ClO_4_ were in accordance with the data from Swager et al.,^34^ where well-defined signals of Co(TPP) (Figure S6a) and no signal of Co(TPP)ClO_4_ protons (spectrum not shown) may be observed. However, upon addition of 10, 20, and 40 equiv of NDMA (by supplier A), there was no change in the NMR spectrum of Co(TPP)ClO_4_. This prompted us to repeat the experiment with Co(TPP), as we concluded that this metalloporphyrin was used in the study in question for the UV–Vis titration. We observed the same phenomenon as in Swager’s report, but again only with a certain NDMA lot (supplier A, Figure S6b). The ^1^H NMR results could not be reproduced with the NDMA lot by supplier B (Figure S6c), as no change in the ^1^H NMR spectra occurred. This is the exact opposite of the observations from the UV–Vis spectroscopy study, where only NDMA from supplier B provided the red shift. Although the newly formed complex has very similar proton signals to metal-free *meso*-tetraphenylporphyrin (TPP), the comparison of the ^1^H NMR spectrum of Co(TPP) and NDMA mixture with the spectrum of TPP (Figure S13) showed some differences (no imine protons in the complex and slightly different positions of Ar–H protons). This indicated that no demetallation of Co(TPP) to TPP took place upon NDMA addition.

The interaction between NDMA and cobalt porphyrins was also studied by Swager et al. using FTIR spectroscopy. Formation of a new signal at 1243 cm^–1^ was observed, which results from shifted NDMA peaks upon binding of NDMA to cobalt metal. This peak was first characterized in iron *meso*-tetraphenylporphyrin complexes with *N*-nitrosamines by Richter-Addo et al.^38−40^ and is also typical for other metals, such as ruthenium and osmium.^39^ The experiment was repeated using Co(TPP)ClO_4_ with NDMA by suppliers A and B (Figure S7). The aforementioned peak was observed with both batches of NDMA. The results thus indicate that the interaction that was described by Swager et al. does take place; however, its UV–Vis and ^1^H NMR manifestations are not as certain.

Throughout our investigations, we have observed the unstable nature of cobalt(III) in CH_2_Cl_2_ solution. Given this information, it might be that Swager et al.^34^ began all experiments using the chemosensor with cobalt(III), which then got reduced to cobalt(II) during the experiments. This should be taken into consideration when interpreting the results of UV–Vis and ^1^H NMR experiments, where the investigated compound was in solution for a longer period of time in comparison to FTIR, where it was applied to NaCl glass as a solid, and only a droplet of NDMA solution was added. Higher stability of bivalent cobalt porphyrins and reduction of tervalent cobalt porphyrins has been observed in the literature,^56^ but a detailed study of cobalt(III) porphyrin stability was out of scope of this paper.

### 2.2 Investigation of the Interaction of Metalloporphyrins with TSNAs

**Figure 4:**
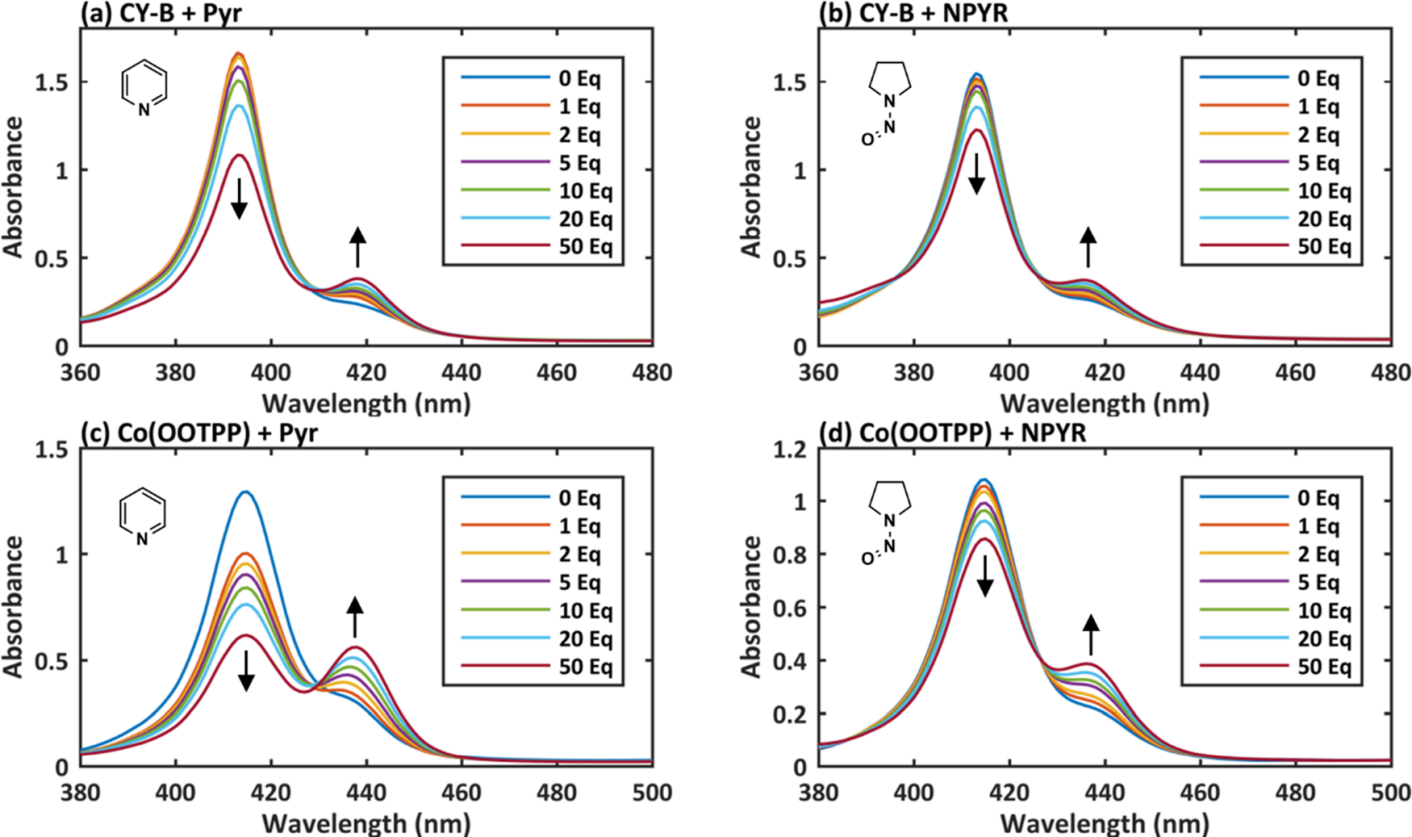
UV–Vis titration curves of metalloporphyrin CY-B with (a) pyridine (Pyr) and (b) *N*-nitrosopyrrolidine (NPYR), and porphyrin F with (c) Pyr and (d) NPYR.

The interaction between cobalt metalloporphyrins and NNN as our model TSNA was studied in more detail to determine the interacting atom of NNN (pyridine-type nitrogen or *N*-nitrosamine moiety). We therefore separately titrated metalloporphyrin CY-B and porphyrin F with pyridine and *N*-nitrosopyrrolidine (NPYR) as individual NNN fragments (Figure 4). Some change in the UV–Vis spectra occurred upon addition of NPYR (Figure 4b,d), but there was also a bathochromic shift upon addition of pyridine (Figure 4a,c), which implicated that the pyridine-type nitrogen may be the interacting atom of NNN. We also evaluated 1-nitroso-2-phenylpyrrolidine (NPP), the benzene analogue of NNN, where the nitrogen atom in the aromatic moiety is replaced with a carbon. A minor red shift in the UV–Vis spectra may be observed with both metalloporphyrin CY-B or porphyrin F (Figure S8). A similar experiment was also carried out with *N*-nitrosopiperidine (NPIP), the nitrosamine fragment of *N*-nitrosoanabasine (NAB), which also produced a small red shift in the UV–Vis spectrum upon addition to metalloporphyrin CY-B or porphyrin F (Figure S8).

**Figure 5:**
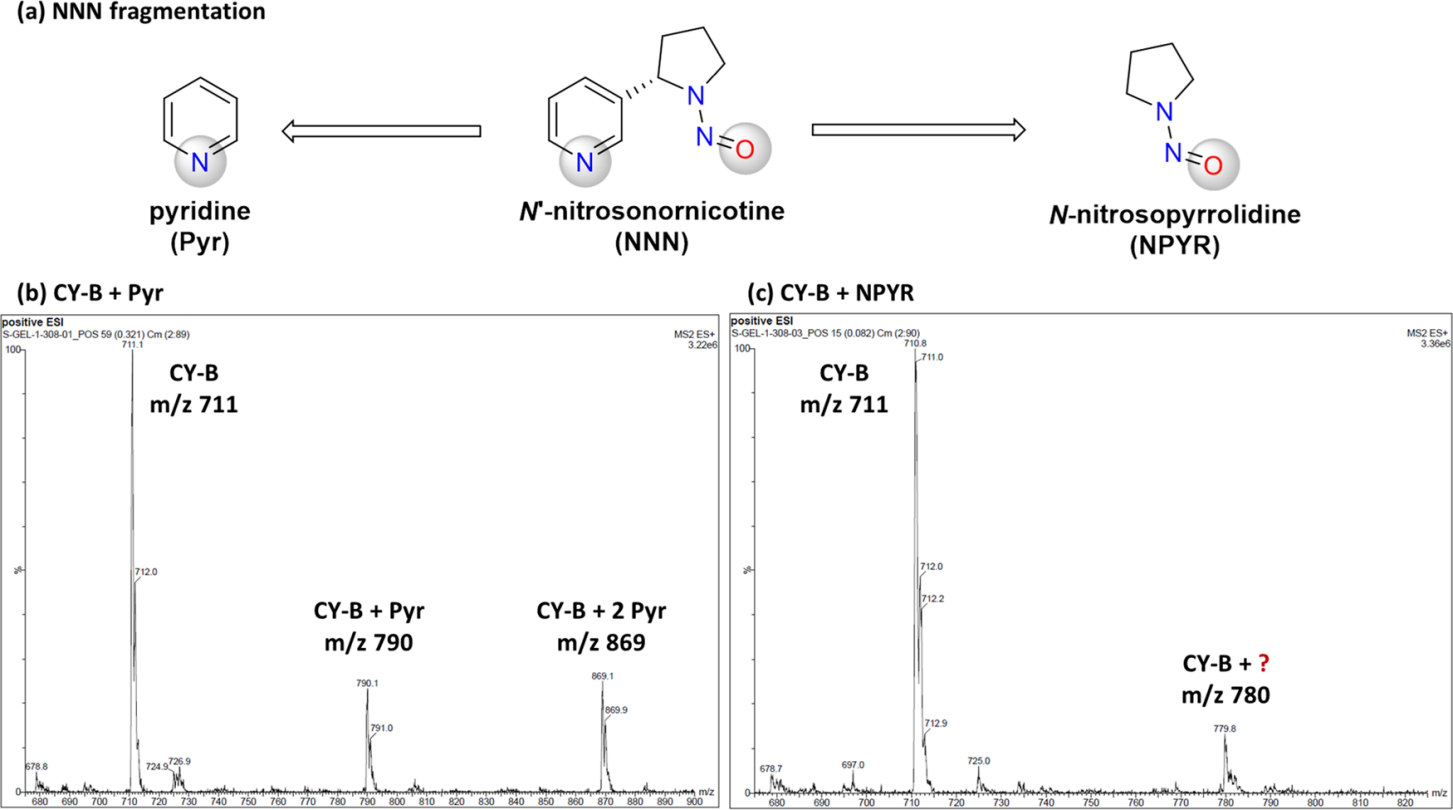
(a) NNN fragmentation to pyridine (Pyr) and *N*-nitrosopyrrolidine (NPYR) for determination of the binding atom. ESI-MS spectrum of metalloporphyrin CY-B with (b) Pyr and (c) NPYR. In panel (c), traces of the complex with unknown ligand with *m*/*z* 69 are seen.

As we were thus not able to determine the interacting atom by UV–Vis spectroscopy, we analyzed the final mixtures of metalloporphyrin CY-B with pyridine and NPYR by mass spectrometry (Figure 5) in a similar manner to the report of Dai and Yu et al.^42^ Interestingly, the 1:1 and 1:2 complexes between metalloporphyrin CY-B and pyridine were formed (*m*/*z* 790 and 869 on Figure 5a), but no metalloporphyrin CY-B complex with NPYR was observed (Figure 5b shows only *m*/*z* 711 of metalloporphyrin CY-B; formation of the complex would be indicated by mass peaks with *m*/*z* 811 and 911). Another peak was detected with *m*/*z* 780, which was likely an indicator of a complex between metalloporphyrin CY-B and a compound with molar mass 69. One possible explanation for this complex could be the presence of 1-pyrroline as an impurity in NPYR since imines are known to be common *N*-nitrosamine degradation products.^4^

While our results by no means dispute the findings of Dai and Yu et al. about metalloporphyrin CY-B/TSNA complex formation and observed red shift in the UV–Vis spectra,^42^ we have shown that the interacting atom is most likely pyridine-type nitrogen and not the postulated *N*-nitrosamine moiety. This further implies that not only TSNAs but all of the major tobacco alkaloids may form such complexes, including nicotine, nornicotine, etc., since their structures contain a pyridine-type nitrogen as well. As we were curious whether two representative tobacco alkaloids nornicotine and anabasine (Figure S9) could also interact via their secondary aliphatic amine moiety, we evaluated the impact of their fragments pyrrolidine and piperidine on the UV–Vis spectra of metalloporphyrin CY-B and porphyrin F (Figure S9). Both produced a red shift in the UV–Vis spectra, indicating binding to the metal center via this way as well, which suggests that the interaction of both studied cobalt porphyrins with various ligands is highly nonspecific. Because the formation of complexes between metalloporphyrins and TSNAs is presented as means of scavenging TSNAs from tobacco smoke,^13,42^ one could therefore argue that the formation of complexes between tobacco alkaloids and metalloporphyrin scavengers in cigarettes may saturate these scavengers and prevent more efficient removal of much more hazardous TSNAs. It is therefore vital to design scavengers in a way that they would be more selective toward TSNAs compared to tobacco alkaloids since the latter will be present in a much larger amount compared to TSNAs.

### 2.3 Investigation of the Interaction of Metalloporphyrins with NDMA

As we were unable to reproduce the red shift upon addition of NDMA to Co(TPP)ClO_4_ presented in the Swager’s report^34^ with all evaluated NDMA samples, which were provided by different vendors, we suspected that the observed red shift in the UV–Vis spectra could be impacted by some synthetic impurities and/or degradation products present only in certain NDMA lots. NDMA is usually synthesized by acid-catalyzed *N*-nitrosation of dimethylamine (DMA) using sodium nitrite, and various references point to a plethora of different acids utilized: acetic acid,^57^ oxalic acid,^58^ hydrochloric acid,^59^ sulfuric acid,^60^ etc., which could remain present in small amounts. Furthermore, NDMA (and other *N*-nitrosamines) is known to form various degradation products depending on the conditions it is exposed to. A detailed schematic presentation on possible degradation pathways and various products formed has been presented thoroughly by Swager et al.^4^ Most final degradation products of NDMA are various acids (formic acid (FA), nitric acid (NA), and unstable nitrous acid) and amines (methylamine (MA) and dimethylamine (DMA)), which means that the likelihood of such compounds present in NDMA in at least trace amounts is relatively high. Different amines, including MA and DMA, are well known in the literature to coordinate with metalloporphyrins as axial ligands.^61^ They also cause UV–Vis spectral changes upon binding, which has already led to development of metalloporphyrins as potential chemosensors for amines.^62,63^ Axial binding of the formate anion to cobalt porphyrin complexes has also been described in the literature.^64^

**Figure 6:**
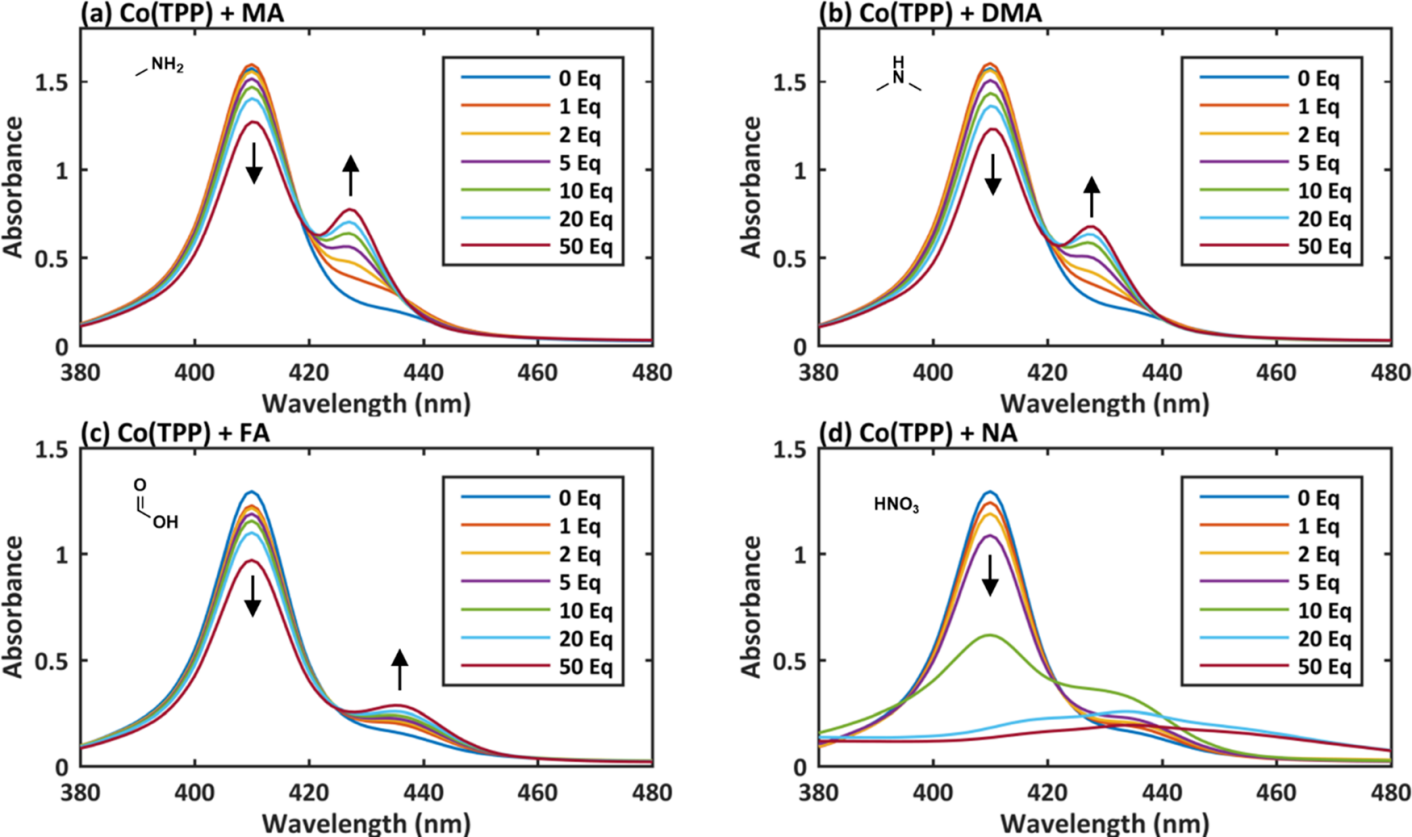
UV–Vis spectra of Co(TPP) titrated with 1–50 equiv of various possible impurities in NDMA: (a) methylamine (MA), (b) dimethylamine (DMA), (c) formic acid (FA), and (d) nitric acid (NA).

**Figure 7:**
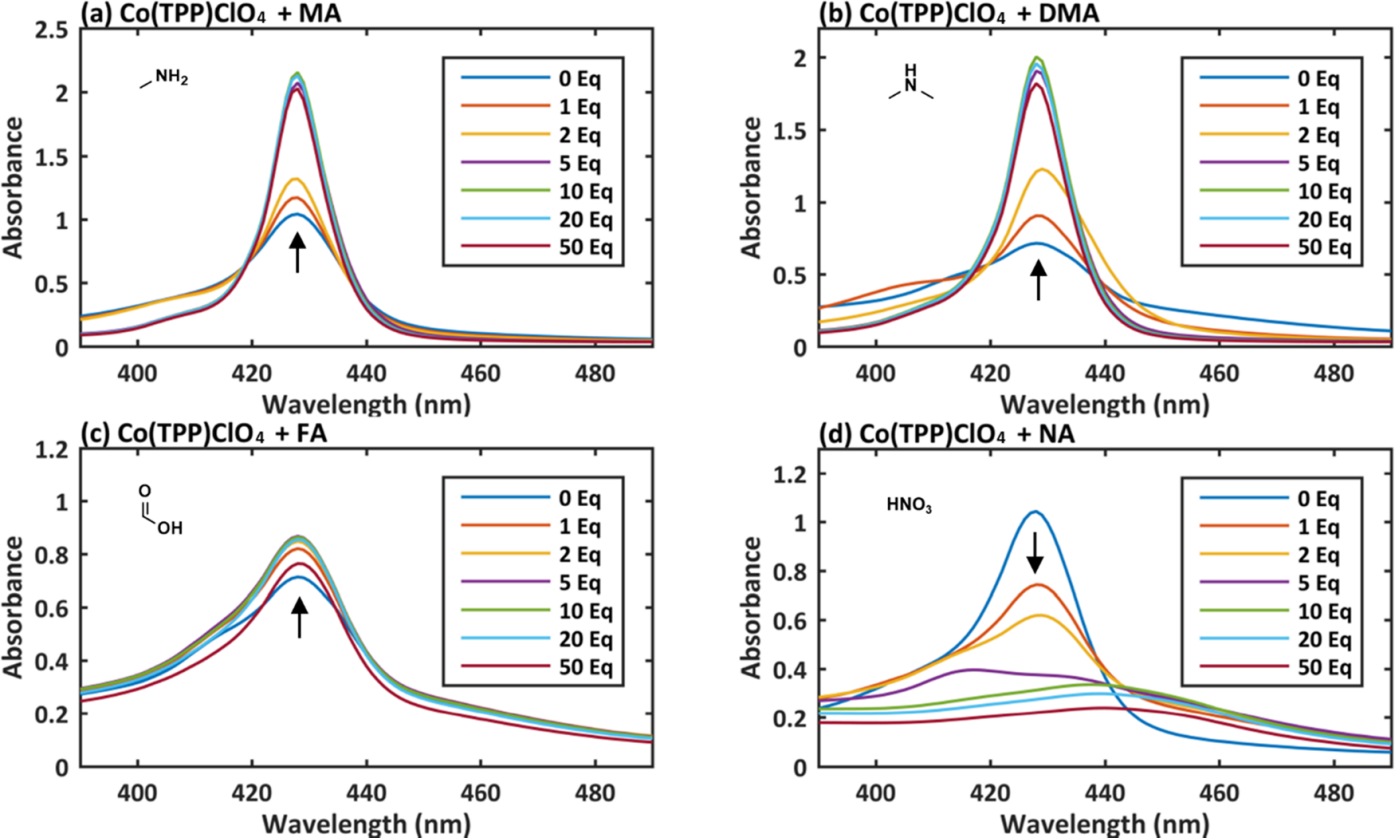
UV–Vis spectra of Co(TPP)ClO_4_ titrated with 1–50 equiv of various possible impurities in NDMA: (a) methylamine (MA), (b) dimethylamine (DMA), (c) formic acid (FA), and (d) nitric acid (NA).

To evaluate the impact of potential NDMA contaminants to the studied cobalt metalloporphyrins’ UV–Vis spectra, we titrated Co(TPP) and Co(TPP)ClO_4_ with MA, DMA, FA, and NA (Figures 6 and 7). Interestingly, the addition of MA (40% aqueous solution), DMA (10% THF solution), and FA led to red shift in the UV–Vis spectrum of Co(TPP) to the same wavelength (428 nm; Figure 6a–c), as observed with one of the evaluated NDMA batches and in the Swager’s study.^34^ Furthermore, NA (added as 65% aqueous solution; Figure 6d), on the other hand, changed the spectrum in a different manner, as the Soret band became much broader and the peak shifted to even higher wavelength (444 nm). The difference in observed changes with both evaluated acids led us to study the impact of two other acids: acetic acid (AA) and trifluoroacetic acid (TFA) (Figures S10c,d and S11c,d). Both led to changes in the UV–Vis spectrum akin to the effect of FA and amines, as opposed to the effect of NA. The impact of two additionally evaluated bases, pyridine and 1,1-dimethylhydrazine (DMH), also produced red shifts to ∼432 nm (Figures S10a,b and S11a,b). Interestingly, the observed changes in Co(TPP)ClO_4_ UV–Vis were identical to the ones seen for Co(TPP), which indicate that the interaction with different ligands is identical for Co(TPP) and Co(TPP)ClO_4_ irrespective of the cobalt oxidation state.

The system thus does not appear to distinguish between amines of different basicity unlike the similar Cr(TPP)Cl, where only weakly basic amines (such as pyridine) act as Lewis bases, forming the Cr(TPP)(Cl)(ligand) complex (with a corresponding red shift in the UV–Vis spectrum). More basic amines, however, behave in a Brønsted base manner to form a [Cr(TPP)Cl(OH)]^−^ complex, which may irreversibly oxidize to the oxido chromium(IV) complex Cr(TPP)(O) (may be recognized using UV–Vis spectroscopy by blue shift of the Soret band).^64^ All amines interact with cobalt metal in Co(TPP) and Co(TPP)ClO_4_ similarly. More diverse interaction types may be observed between metalloporphyrin and different acids. One may see that carboxylic acids (AA, FA, TFA) interact similarly to amines, while strong inorganic acids (NA) provide a completely different interaction as evidenced by UV–Vis spectra.

**Figure 8:**
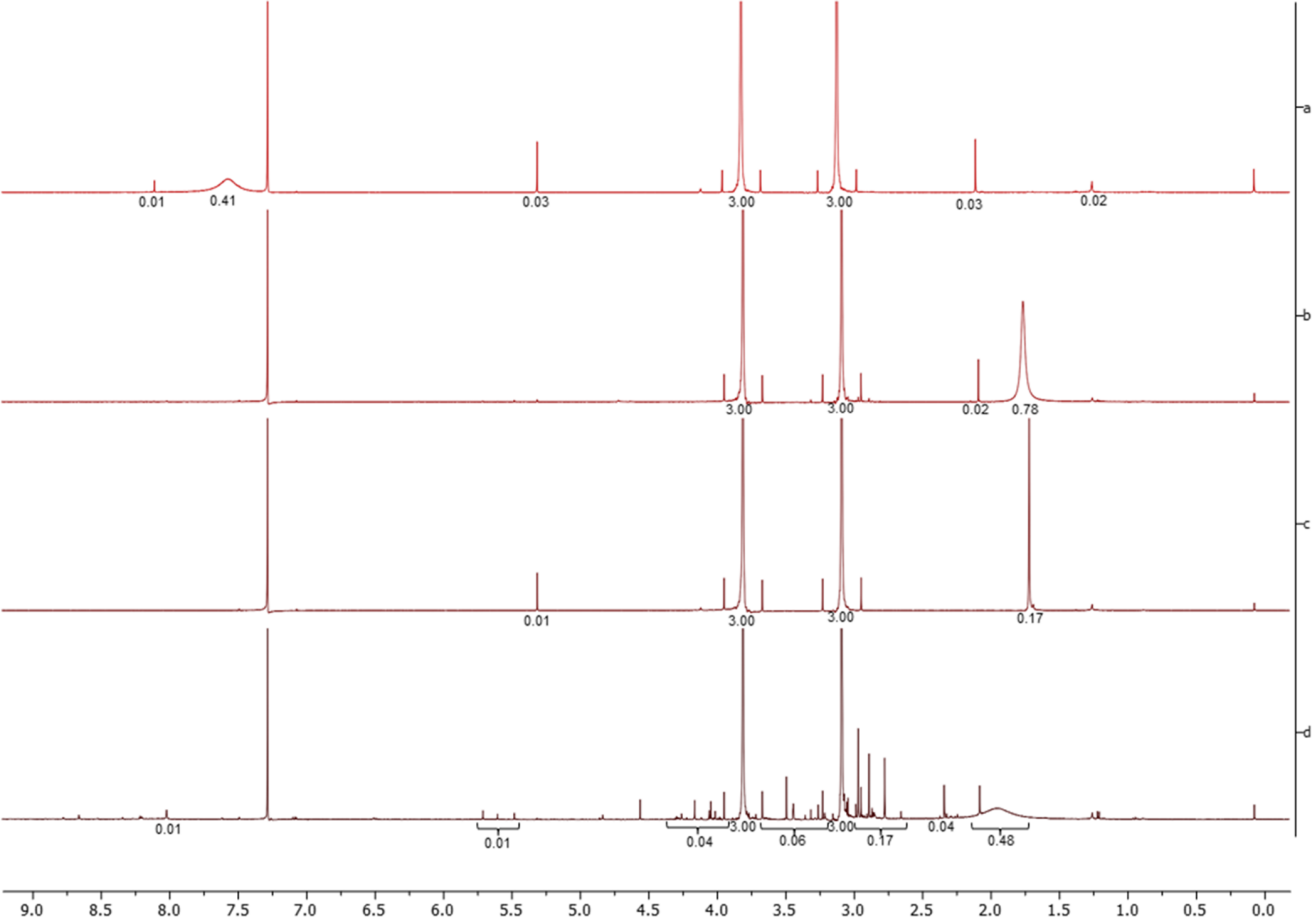
^1^H NMR spectra (CDCl_3_) of NDMA by (a) supplier A, (b) supplier B, (c) supplier A (neutralized with solid K_2_CO_3_), and (d) supplier B (photodegraded).

NDMA itself is not very acidic and although *N*-nitrosamines may be deprotonated at the α position, very strong bases such as LDA are required.^65^ One of the key pieces of evidence that NDMA might contain acidic impurities is the water signal in the ^1^H NMR spectra of pure NDMA (Figure 8a,b). The signal is not present as a sharp singlet at 1.56 ppm^66^ but is shifted to a lower field and presented as a broad singlet. In the NDMA sample by supplier A, the water signal may be observed at 7.57 ppm and in the NDMA sample by supplier B at 1.75 ppm. A shift of water signal to the lower field due to lower pH has been described in the literature.^67^ To evaluate if NDMA was contaminated with acidic impurities, pure NDMA (by supplier A) was stirred over solid K_2_CO_3_ overnight and then dissolved in CH_2_Cl_2_ (0.2 mg/mL). In the ^1^H NMR spectrum of the obtained sample (Figure 8c), the water signal is also sharper and located at 1.71 ppm, which resembles the sample by supplier B as well as literature data.^66^ We have also analyzed all three samples (NDMA by supplier A, NDMA by supplier B, and NDMA by supplier A that was neutralized with K_2_CO_3_) by FTIR spectroscopy (Figure S12). An additional peak at 952 cm^–1^ and additional splitting of the peak at 686 cm^–1^ were found in the sample that gave a red shift in the UV–Vis spectrum of TPP (supplier A) compared to the other two (supplier B, K_2_CO_3_-treated supplier A). Furthermore, we also wanted to evaluate the possibility of NDMA photodegradation, so we exposed one NDMA sample (by supplier B) to daylight. A 0.2 mg/mL solution in CH_2_Cl_2_ was left on the window shelf for 3 days. Comparison of ^1^H NMR spectra before and after the exposure shows that the quality of NDMA worsens considerably by exposure to daylight (Figure 8d). Several new peaks may be observed and the water signal broadens and gets shifted downfield to 1.95 ppm.

### 2.4 Investigation of Interaction of Nitrosamines with Metal-Free Porphyrins and Phthalocyanines

**Figure 9:**
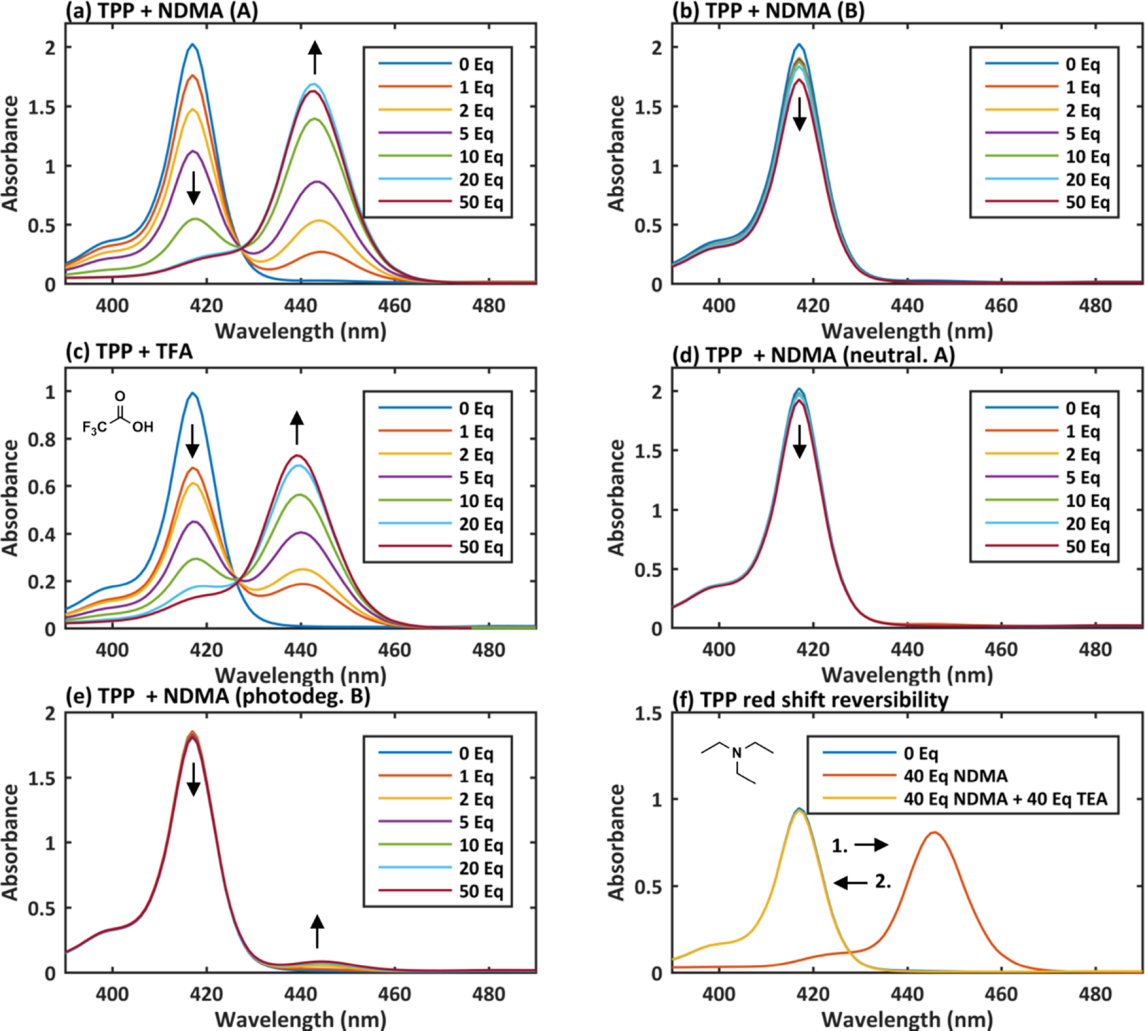
Interaction of different batches of NDMA with TPP in comparison with the interaction of TFA with TPP as studied by UV–Vis spectroscopy is shown. To reverse the protonation-caused red shift, UV–Vis spectra after TEA addition are also shown. UV–Vis spectra of TPP titrated with 1–50 equiv of (a) NDMA by supplier A, (b) NDMA by supplier B, (c) NDMA by supplier A, (c) TFA, (d) NDMA by supplier B (K_2_CO_3_-treated), (e) NDMA by supplier B (photodegraded), and (f) reversed spectra with TEA addition.

During our investigation, we have also studied several metal-free porphyrins (as starting materials for metallation) by UV–Vis spectroscopy. Surprisingly, we observed that upon titration of TPP with NDMA (40 equiv) by supplier A, the porphyrin’s Soret band in the UV–Vis spectrum is also red-shifted (Figure 9a). This interaction has not been previously known in the literature, and due to the lack of metal center to which the *N*-nitrosamine group could coordinate to produce a bathochromic shift, we were highly interested in discovering the origin of this phenomenon. We therefore analyzed the interaction of NDMA (by supplier A) and TPP by ^1^H NMR spectroscopy (Figure S13). A distinct change of the location of aromatic protons of TPP may be observed in the spectrum upon NDMA addition and surprisingly also in both the position and number of the pyrrole NH protons. Two highly shielded protons at −2.8 ppm disappear, and four protons appear at −0.6 ppm with the addition of NDMA. This observation could indicate the protonation of TPP by acidic impurities present in NDMA (by supplier A), which led us to investigate deeper into the physicochemical properties of porphyrins.

Porphyrins have basic properties, which have been widely studied in the literature.^68−70^ They may bind two protons with the imine-type pyrrole nitrogens, and nonprotonated and protonated species are known to differ in their optical properties. These may be observed even visually through color change after addition of an acid to porphyrin solution as well as by UV–Vis spectroscopy. A bathochromic shift of the Soret band in the UV–Vis spectra is observed upon protonation of porphyrins. Similar to the metalloporhyrin/ligand interaction, the Q band region also changes, as several bands disappear and a new bathochromically shifted one appears. This also explains the visual color change of the TPP solution from purple to green.^71−73^ To compare the impact of *N*-nitrosamines and acids on porphyrin UV–Vis and ^1^H NMR spectra, we evaluated the interaction between trifluoroacetic acid (TFA) and TPP ourselves (Figures 9c and S13d). The obtained spectra are identical to the ones produced by the TPP/NDMA (by supplier A) interaction (Figures 9a and S13b) and are also in agreement with the spectra of protonated TPP in the literature.^68,70^ Due to the low acidity of α-carbon atoms of *N*-nitrosamines, this serves as a further confirmation of the presence of acidic impurities in *N*-nitrosamines, which may cause a red shift in the UV–Vis spectra of metal-free porphyrins. To prove the reversibility of the protonation, an excess of triethylamine (TEA) as a base was added to red-shifted solution of TPP and NDMA, and the initial Soret band at 417 nm was obtained again (Figure 9f). To confirm that the interaction between TPP and NDMA by supplier A is due to the presence of acidic impurities, an experiment was conducted with base-stirred NDMA by supplier A. A pure compound was mixed with K_2_CO_3_ overnight and 0.2 mg/mL solution in CH_2_Cl_2_ was prepared. No red shift was observed upon addition to a TPP solution, confirming the hypothesis (Figure 9d).

Furthermore, we evaluated the TPP/NDMA (by supplier B) interaction (Figures 9b and S13b). Interestingly, NDMA by supplier B behaved completely differently than the one by supplier A; neither red shift in the UV–Vis spectrum nor protonation in the ^1^H NMR spectrum was observed. Both results are in agreement with our hypothesis that NDMA by supplier A contains acidic impurities. Exposure of NDMA by supplier B to light and its addition to TPP led to a slight red shift in the UV–Vis spectrum (Figure 9e), indicating the formation of acidic degradants.

Interestingly, a comparison of the wavelength of the red-shifted Soret band of TPP upon protonation (444 nm) matches the red-shifted Soret band in the UV–Vis spectrum of Co(TPP) and Co(TPP)ClO_4_ upon addition of stronger acids as shown in Figures 6d and 7d with NA, although it is not as broad. It is therefore possible that strong acids could at least partially demetallate the studied cobalt porphyrins and release protonated TPP, although it is likely that other reactions take place as well. Metalloporphyrins are known to be acid-labile depending on the porphyrin structure and nature of the central metal and acidic demetallation can be in fact used to compare the stability of different metalloporphyrins.^74^ To assess whether *N*-nitrosamines may cause demetallation as well, we added NDMA by supplier A (shown to contain acidic impurities) to solutions of various metal *meso*-tetraphenylporphyrins in CH_2_Cl_2_. We have observed demetallation of Cd(II), Er(III), Eu(III), Gd(III), and Pb(II) *meso*-tetraphenylporphyrins, as the metalloporphyrins’ Soret bands shifted to 444 nm, which is indicative of protonated TPP (Figure S14).

A related group of compounds to porphyrins are (metallo)phthalocyanines. Upon our study, we have also evaluated cobalt(II) octabutoxyphthalocyanine (Co(OBu)_8_Pc) and observed that impure NDMA addition also leads to distinct changes in its UV–Vis spectrum (general structure of metallophthalocyanines and UV–Vis spectra of Co(OBu)_8_Pc titration with NDMA are shown in Figure S15a). Metallophthalocyanines are also basic and may bind four protons with their imine-type nitrogen atoms at the *meso* positions. In contrast to porphyrins, phthalocyanines do not lose their basicity upon metal insertion, as the nitrogens at the *meso* positions remain available for protonation. Basic properties can even be enhanced by the presence of a metal ion, e.g., Zn(OBu)_8_Pc compared to H_2_(OBu)_8_Pc.^75^ Protonation of (metallo)phthalocyanines leads to very distinct bathochromic shifts of the main Q band for each sequential protonation, with the band of the tetraprotonated species being the most bathochromically shifted. This is especially typical for (metallo)phthalocyanines that form an intramolecular hydrogen bond with hydrogen bond acceptors at peripheral positions upon protonation.^75^ By observing the titration curves in Figure S15b, one can see that NDMA (by supplier A) addition to Co(OBu)_8_Pc mimics the addition of TFA to Zn(OBu)_8_Pc as in Fukuzumi’s study.^75^

### 2.5 Other Evidence in the UV–Vis Spectra for Presence of Red Shift-Producing Impurities in *N*-Nitrosamines

Solid-phase extraction (SPE) is a common isolation method for NDMA from various matrices before further analysis.^76^ During our development of the SPE method to isolate NDMA from aqueous samples, we have been constantly challenged by the lack of porphyrin/phthalocyanine red shift upon UV–Vis analysis of isolated NDMA. Its presence in the extract was confirmed by LC-MS analysis. Our initial explanation was that certain trace impurities were leaching from the coconut charcoal, used for SPE, and blocking the red shift-producing interaction between NDMA and porphyrin. However, similar issues were observed by both continuous and batch liquid–liquid extraction using CH_2_Cl_2_ as the organic extraction solvent. However, the addition of fresh NDMA solution to the final extract produced a red shift in the UV–Vis spectra. Upon confirming the origin of porphyrin/phthalocyanine red shift, we could explain the observed UV–Vis behavior of extracted samples. Trace acidic impurities were not extracted on solid charcoal and/or into CH_2_Cl_2_ and were therefore absent in the final extract. With these observations, we have therefore inadvertently confirmed that the bathochromic shift is not the result of NDMA.

## 3 Conclusions

In summary, we have conducted extensive re-evaluation of previous studies that highlighted the interaction between *N*-nitrosamines and metalloporphyrins as a potential optical sensing option using UV–Vis spectroscopy. Our results indicate that several other chemical species that can be present in *N*-nitrosamines (including *N*-nitrosamine degradation products) produce optical changes that were previously associated only with metalloporphyrin/*N*-nitrosamine interactions. Moreover, we have demonstrated that in the case of TSNAs, other functional groups present in the analytes react with metalloporphyrin and provide optical changes in UV–Vis spectra that were previously ascribed to the binding of the *N*-nitrosamine moiety to the metal center of metalloporphyrins. The major findings may therefore be summarized in the following key points:

- It is premature to consider cobalt-based metalloporphyrins as suitable probes for selective UV–Vis detection of *N*-nitrosamine moiety-containing species.
- Caution should be taken when interpreting the observed red shifts in UV–Vis spectra when metalloporphyrins are considered as chemosensors, especially when used with samples of lower chemical purity or analytes bearing multiple functional groups that are capable of coordination to the metal center in metalloporphyrin.
- Further research in the area of metalloporphyrin chemosensors should put utmost importance in evaluation of selectivity and robustness of metalloporphyrin-based chemosensors.

## 4 Materials and Methods

### 4.1 Chemicals and Reagents

*Meso*-tetra(4-octyloxyphenyl)porphyrin was purchased from PorphyChem (Dijon, France). MgSO_4_, Co(OAc)_2_·4H_2_O, and MnSO_4_·H_2_O were purchased from Sigma-Aldrich (St. Louis, MO). (*Meso*-tetraphenylporphyrinato)cobalt(II) was purchased from Strem chemicals, Inc. (Newburyport, MA). 5,10,15,20-Tetraphenyl-21*H*,23*H*-porphin was purchased from Sigma-Aldrich (St. Louis, MO). All chemicals were of reagent grade and were used as supplied.

*Meso*-tetra(4-octylphenyl)porphyrin was synthesized, according to the literature procedure.^77^ Metalloporphyrin CY-B was synthesized, according to the published literature procedure from protoporphyrin IX.^42^ (*Meso*-tetra(4-octyloxyphenyl)porphinato)cobalt(II), (*meso*-tetra(4-octylphenyl)porphinato)cobalt(II), (*meso*-tetra(4-octyloxyphenyl)porphinato)manganate(II), and (*meso*-tetra(4-octyloxyphenyl)porphinato)manganate(II) were synthesized, according to the published literature procedure.^46^

*N*,*N*-dimethylformamide was of reagent grade and purchased from BASF SE (Ludwigshafen, Germany). CH_2_Cl_2_, methanol, and acetonitrile were of spectroscopic grade and were purchased from Supelco (Bellefonte, PA). Pyridine (anhydrous, 99.8%), nitric acid (65% water solution), perchloric acid (70% water solution), and methylamine (40% water solution) were purchased from Sigma-Aldrich (St. Louis, MO). Formic acid (99%) was purchased from Carlo Erba Reagents (Cornaredo, Milano, Italy). Dimethylamine (10% THF solution) was purchased from TCI (Boereveldseweg, Zwijndrecht, Belgium). Deuterated dichloromethane (CD_2_Cl_2_) and chloroform (CDCl_3_) were purchased from Sigma-Aldrich (St. Louis, MO).

NDMA was purchased from Clearsynth (Villeurbanne, France; supplier A) and Sigma-Aldrich (St. Louis, MO; supplier B). NPIP and NPYR were purchased from Agilent (Zug, Switzerland). NNN, NAB, NAT, and NNK were purchased from Sigma-Aldrich (St. Louis, MO). 1-Nitroso-2-phenylpyrrolidine was purchased from ChemSpace (Riga, Latvia).

### 4.2 Equipment and Software

UV–Vis absorption spectra were recorded on a Cary 3500 Series UV–Vis spectrophotometer (Agilent, Santa Clara, CA), using a 10 mm pathlength quartz cuvette (Starna Scientific Ltd.) at room temperature (25 °C).

ATR-FTIR spectra were recorded on a Nicolet iS50 FTIR spectrometer (Thermo Fisher Scientific, Waltham, MA) using a single reflection diamond ATR cell.

All ^1^H NMR measurements were carried out on a Bruker Avance III spectrometer (Bruker Biospin, Rheinstetten, Germany) operating at 500 MHz. The spectrometer was equipped with a 5 mm BBO, Z-gradient probe. Spectra were acquired and processed using Bruker TopSpin software, version 3.1. Spectra were recorded in CD_2_Cl_2_ or CDCl_3_. ^1^H NMR chemical shifts are reported in parts per million (δ) relative to solvent resonances, which served as internal standards (CD_2_Cl_2_, 5.32 ppm; CDCl_3_, 7.26 ppm). Data are reported as follows: chemical shift, multiplicity (s = singlet, br s = broad singlet, d = doublet, t = triplet, q = quartet, m = multiplet), coupling constants (*J*, in hertz), and integration.

UV–Vis spectra were drawn in Matlab 2015b (Mathworks, MA). Structural formulas were drawn in ChemDraw Professional 19.1, which is part of ChemOffice 2019 package (PerkinElmer Inc., MA).

Electrospray ionization mass spectra (ESI-MS) were recorded in positive ion mode on a Waters Xevo triple quadrupole mass spectrometer (Milford, MA). The methanolic sample solutions were infused into the MS at 50 μL/min flow along with a 300 μL/min flow of mobile phase (acetonitrile/0.1% (v/v) formic acid in water = 6/4 (v/v)). The ESI parameters were as follows: capillary voltage 1.5 kV, cone voltage 17 V, desolvation temperature 550 °C, desolvation and cone gas flows −800 and 20 L/h. The positive ESI mass spectra were acquired in the mass range from *m*/*z* 80 to 2000. The analyte ions were detected as protonated molecules [M + H]^+^.

## Supporting Information Available

Figure S1: UV–Vis spectra of different possibilities for porphyrin F structure; Figure S2: UV–Vis spectra of *meso*-tetra-(4-octoxylphenyl) cobalt porphyrin (first of the two possible porphyrin F structures) titrated with TSNAs; Figure S3: UV–Vis spectra of meso-tetra-(4-octylphenyl) cobalt porphyrin (second of the two possible porphyrin F structures) titrated with TSNAs; Figure S4: UV–Vis spectra of metalloporphyrin CY-B titrated with TSNAs; Figure S5: ESI-MS spectra of metalloporphyrin CY-B titrated with TSNAs; Figure S6: ^1^H NMR spectra of Co(TPP) with NDMA; Figure S7: FTIR spectra of NDMA and Co(TPP)ClO_4_ with NDMA; Figure S8: UV–Vis spectra of metalloporphyrin CY-B and porphyrin F with TSNA analogues; Figure S9: UV–Vis spectra of metalloporphyrin CY-B and porphyrin F with secondary amines; Figure S10: UV–Vis spectra of Co(TPP) with various bases and acids; Figure S11: UV–Vis spectra of Co(TPP)ClO_4_ with various bases and acids; Figure S12: FTIR spectra of different NDMA batches; Figure S13: ^1^H NMR spectra of TPP with NDMA and TFA; Figure S14: UV–Vis spectra of selected acid-labile metalloporphyrins before and after the addition of NDMA by supplier A; Figure S15: general structure of (metallo)phthalocyanines and UV–Vis spectra of Co(OBu)_8_Pc with NDMA (PDF)

## References

[ref1] Tricker A. R.; Preussmann R. Carcinogenic *N*-nitrosamines in the diet: occurrence, formation, mechanisms and carcinogenic potential. Mutat. Res. 1991, 259, 277–289. 10.1016/0165-1218(91)90123-4. 2017213

[ref2] Kroeger-Koepke M. B.; Koepke S. R.; McClusky G. A.; Magee P. N.; Michejda C. J. alpha-Hydroxylation pathway in the in vitro metabolism of carcinogenic nitrosamines: N-nitrosodimethylamine and *N*-nitroso-*N*-methylaniline. Proc. Natl. Acad. Sci. U.S.A. 1981, 78, 6489–6493. 10.1073/pnas.78.10.6489. 6947239 PMC349065

[ref3] Guengerich F. P.; Shimada T. Oxidation of toxic and carcinogenic chemicals by human cytochrome P-450 enzymes. Chem. Res. Toxicol. 1991, 4, 391–407. 10.1021/tx00022a001. 1912325

[ref4] Beard J. C.; Swager T. M. An Organic Chemist’s Guide to *N*-Nitrosamines: Their Structure, Reactivity, and Role as Contaminants. J. Org. Chem. 2021, 86, 2037–2057. 10.1021/acs.joc.0c02774. 33474939 PMC7885798

[ref5] Li Y.; Hecht S. S. Metabolic Activation and DNA Interactions of Carcinogenic *N*-Nitrosamines to Which Humans Are Commonly Exposed. Int. J. Mol. Sci. 2022, 23, 4559 10.3390/ijms23094559. 35562949 PMC9105260

[ref6] Li Y.; Hecht S. S. Metabolism and DNA Adduct Formation of Tobacco-Specific *N*-Nitrosamines. Int. J. Mol. Sci. 2022, 23, 5109 10.3390/ijms23095109. 35563500 PMC9104174

[ref7] Scanlan R. A. Formation and occurrence of nitrosamines in food. Cancer Res. 1983, 43, 2435s–2440s. 6831466

[ref8] Bharate S. S. Critical Analysis of Drug Product Recalls due to Nitrosamine Impurities. J. Med. Chem. 2021, 64, 2923–2936. 10.1021/acs.jmedchem.0c02120. 33706513

[ref9] Challis B. C.; Trew D. F.; Guthrie W. G.; Roperr D. V. Reduction of nitrosamines in cosmetic products. Int. J. Cosmet. Sci. 1995, 17, 119–131. 10.1111/j.1467-2494.1995.tb00115.x. 19245496

[ref10] Fajen J. M.; Carson G. A.; Rounbehler D. P.; Fan T. Y.; Vita R.; Goff U. E.; Wolf M. H.; Edwards G. S.; Fine D. H.; Reinhold V.; Biemann K. *N*-Nitrosamines in the Rubber and Tire Industry. Science 1979, 205, 1262–1264. 10.1126/science.472741. 472741

[ref11] Hecht S. S.; Hoffmann D. Tobacco-specific nitrosamines, an important group of carcinogens in tobacco and tobacco smoke. Carcinogenesis 1988, 9, 875–884. 10.1093/carcin/9.6.875. 3286030

[ref12] Nawrocki J.; Andrzejewski P. Nitrosamines and water. J. Hazard. Mater. 2011, 189, 1–18. 10.1016/j.jhazmat.2011.02.005. 21353742

[ref13] Wang C.; Dai Y.; Feng G.; He R.; Yang W.; Li D.; Zhou X.; Zhu L.; Tan L. Addition of Porphyrins to Cigarette Filters to Reduce the Levels of Benzo[*a*]pyrene (B[a]P) and Tobacco-Specific *N*-Nitrosamines (TSNAs) in Mainstream Cigarette Smoke. J. Agric. Food Chem. 2011, 59, 7172–7177. 10.1021/jf200966p. 21662235

[ref14] Edwards S. H.; Hassink M. D.; Taylor K. M.; Watson C. H.; Kuklenyik P.; Kimbrell B.; Wang L.; Chen P.; Valentín-Blasini L. Tobacco-Specific Nitrosamines in the Tobacco and Mainstream Smoke of Commercial Little Cigars. Chem. Res. Toxicol. 2021, 34, 1034–1045. 10.1021/acs.chemrestox.0c00367. 33667338

[ref15] aSchmidtsdorff S.; Neumann J.; Schmidt A. H.; Parr M. K. Risk assessment for nitrosated pharmaceuticals: A future perspective in drug development. Arch. Pharm. 2022, 355, 2100435 10.1002/ardp.202100435. 35088435

[ref16] European Medicines Agency. ICH Guideline M7(R1) on Assessment and Control of DNA Reactive (Mutagenic) Impurities in Pharmaceuticals to Limit Potential Carcinogenic Risk. EMA/CHMP/ICH/83812/2013. https://www.ema.europa.eu/en/documents/scientific-guideline/ich-guideline-m7r1-assessment-control-dna-reactive-mutagenic-impurities-pharmaceuticals-limit_en.pdf (accessed August 11, 2022).

[ref17] Dobo K. L.; Kenyon M. O.; Dirat O.; Engel M.; Fleetwood A.; Martin M.; Mattano S.; Musso A.; McWilliams J. C.; Papanikolaou A.; Parris P.; Whritenour J.; Yu S.; Kalgutkar A. S. Practical and science-based strategy for establishing acceptable intakes for drug product *N*-nitrosamine impurities. Chem. Res. Toxicol. 2022, 35, 475–489. 10.1021/acs.chemrestox.1c00369. 35212515 PMC8941624

[ref18] Elder D. P.; Johnsonb G. E.; Snodin D. J. Tolerability of risk: A commentary on the nitrosamine contamination issue. J. Pharm. Sci. 2021, 110, 2311–2328. 10.1016/j.xphs.2021.02.028. 33705731

[ref19] Tuesuwan B.; Vongsutilers V. Nitrosamine contamination in pharmaceuticals: threat, impact, and control. J. Pharm. Sci. 2021, 110, 3118–3128. 10.1016/j.xphs.2021.04.021. 33989680

[ref20] Li K.; Ricker K.; Tsai F. C.; Hsieh C. J.; Osborne G.; Sun M.; Marder M. E.; Elmore S.; Schmitz R.; Sandy M. S. Estimated cancer risks associated with nitrosamine contamination in commonly used medications. Int. J. Environ. Res. Public Health 2021, 18, 9465 10.3390/ijerph18189465. 34574388 PMC8467924

[ref21] Sörgel F.; Kinzig M.; Abdel-Tawab M.; Bidmon C.; Schreiber A.; Ermel S.; Wohlfart J.; Besa A.; Scherf-Clavel O.; Holzgrabe U. The contamination of valsartan and other sartans, part 1: New findings. J. Pharm. Biomed. Anal. 2019, 172, 395–405. 10.1016/j.jpba.2019.05.022. 31122801

[ref22] James M.; Edge T. Low-Level Determination of Mutagenic Nitrosamine Impurities in Drug Substances by LC–MS/MS. LC·GC Eur. 2021, 34, 267–276.

[ref23] Nagendla N. K.; Shaik H.; Subrahanyam S. B.; Godugu D.; Mudiam M. K. R. Development, validation, and estimation of measurement uncertainty for the quantitative determination of nitrosamines in Sartan drugs using liquid chromatography-atmospheric pressure chemical ionization-tandem mass spectrometry. J. Chromatogr. Open 2022, 2, 100053 10.1016/j.jcoa.2022.100053.

[ref24] Asare S. O.; Hoskins J. N.; Blessing R. A.; Hertzler R. L. Mass spectrometry based fragmentation patterns of nitrosamine compounds. Rapid Commun. Mass Spectrom. 2022, 36, e9261 10.1002/rcm.9261. 35088453

[ref25] Shaik K. M.; Sarmah B.; Wadekar G. S.; Kumar P. Regulatory Updates and analytical methodologies for nitrosamine impurities detection in sartans, ranitidine, nizatidine, and metformin along with sample preparation techniques. Crit. Rev. Anal. Chem. 2022, 52, 53–71. 10.1080/10408347.2020.1788375. 32691615

[ref26] Wang Q.; Liu Z.; Liu Y.; Chen H. Absolute quantitation of N-nitrosamines by coulometric mass spectrometry without using standards. J. Am. Soc. Mass Spectrom. 2022, 33, 875–884. 10.1021/jasms.2c00064. 35446584 PMC9119692

[ref27] Mallavarapu R.; Katari N. K.; Siddhani V. K.; Marisetti V. M.; Rekulapally V. K.; Vyas G.; Jonnalagadda S. B. Development and validation of rapid ultra high performance liquid chromatography with tandem mass spectroscopic method for the quantification of *N*-Nitrosodimethyl amine and *N*-Nitrosodiethyl amine in sitagliptin and metformin hydrochloride immediate and extended-release formulations. J. Sep. Sci. 2022, 45, 3067–3081. 10.1002/jssc.202200226. 35771715

[ref28] U.S. Food and Drug Administration. Control of Nitrosamine Impurities in Human Drugs. https://www.fda.gov/media/141720/download (accessed August 11, 2022).

[ref29] Coffacci L.; Codognoto L.; Fleuri L. F.; Lima G. P. P.; Pedrosa V. A. Determination of Total Nitrosamines in Vegetables Cultivated Organic and Conventional Using Diamond Electrode. Food Anal. Methods 2013, 6, 1122–1127. 10.1007/s12161-012-9518-z.

[ref30] Minami T.; Esipenko N. A.; Zhang B.; Kozelkova M. E.; Isaacs L.; Nishiyabu R.; Kubo Y.; Anzenbacher P. Supramolecular Sensor for Cancer-Associated Nitrosamines. J. Am. Chem. Soc. 2012, 134, 20021–20024. 10.1021/ja3102192. 23194337

[ref31] Cetó X.; Saint C. P.; Chow C. W. K.; Voelcker N. H.; Prieto-Simón B. Electrochemical detection of *N*-nitrosodimethylamine using a molecular imprinted polymer. Sens. Actuators, B 2016, 237, 613–620. 10.1016/j.snb.2016.06.136.

[ref32] Breider F.; Von Gunten U. Quantification of Total *N*-Nitrosamine Concentrations in Aqueous Samples via UV-Photolysis and Chemiluminescence Detection of Nitric Oxide. Anal. Chem. 2017, 89, 1574–1582. 10.1021/acs.analchem.6b03595. 27989108

[ref33] Majumdar S.; Thakur D.; Chowdhury D. DNA Carbon-Nanodots based Electrochemical Biosensor for Detection of Mutagenic Nitrosamines. ACS Appl. Bio Mater. 2020, 3, 1796–1803. 10.1021/acsabm.0c00073. 35021669

[ref34] He M.; Croy R. G.; Essigmann J. M.; Swager T. M. Chemiresistive Carbon Nanotube Sensors for *N*-nitrosodialkylamines. ACS Sens. 2019, 4, 2819–2824. 10.1021/acssensors.9b01532. 31573183 PMC6939299

[ref35] Lu R.-Q.; Yuan W.; Croy R. G.; Essigmann J. M.; Swager T. M. Metallocalix[4]arene Polymers for Gravimetric Detection of *N*-Nitrosodialkylamines. J. Am. Chem. Soc. 2021, 143, 19809–19815. 10.1021/jacs.1c08739. 34793165 PMC8811785

[ref36] Wang Q.; Liu Z.; Liu Y.; Chen H. Absolute Quantitation of *N*-Nitrosamines by Coulometric Mass Spectrometry without Using Standards. J. Am. Soc. Mass Spectrom. 2022, 33, 875–884. 10.1021/jasms.2c00064. 35446584 PMC9119692

[ref37] Appel K. E.; Ruf H. H.; Mahr B.; Schwarz M.; Rickart R.; Kunz W. Binding of nitrosamines to cytochrome P-450 of liver microsomes. Chem.–Biol. Interact. 1979, 28, 17–33. 10.1016/0009-2797(79)90111-X. 227615

[ref38] Yi G.-B.; Khan M. A.; Richter-Addo G. B. The First Metalloporphyrin Nitrosamine Complex: Bis(diethylnitrosamine)(meso-tetraphenylporphyrinato)iron(III) perchlorate. J. Am. Chem. Soc. 1995, 117, 7850–7851. 10.1021/ja00134a048.

[ref39] Chen L.; Yi G.-B.; Wang L.-S.; Dharmawardana U. R.; Dart A. C.; Khan M. A.; Richter-Addo G. B. Synthesis, Characterization, and Molecular Structures of Diethylnitrosamine Metalloporphyrin Complexes of Iron, Ruthenium, and Osmium. Inorg. Chem. 1998, 37, 4677–4688. 10.1021/ic9801591. 11670621

[ref40] Xu N.; Goodrich L. E.; Lehnert N.; Powell D. R.; Richter-Addo G. B. Five- and Six-Coordinate Adducts of Nitrosamines with Ferric Porphyrins: Structural Models for the Type II Interactions of Nitrosamines with Ferric Cytochrome P450. Inorg. Chem. 2010, 49, 4405–4419. 10.1021/ic901751z. 20392126 PMC2896561

[ref41] Lee J.; Chen L.; West A. H.; Richter-Addo G. B. Interaction of Organic Nitroso Compounds with Metals. Chem. Rev. 2002, 102, 1019–1066. 10.1021/cr0000731. 11942786

[ref42] Tao F.; Dai Y.; Wang C.; Feng G.; Li D.; Ma K.; Zhu L.; Tan L.; Yu X. The interaction of a cobalt porphyrin with cancer-associated nitrosamines. Bioorg. Chem. 2014, 56, 67–74. 10.1016/j.bioorg.2014.07.007. 25123542

[ref43] Qi Z.-L.; Cheng Y.-H.; Xu Z.; Chen M.-L. Recent Advances in Porphyrin-Based Materials for Metal Ions Detection. Int. J. Mol. Sci. 2020, 21, 5839 10.3390/ijms21165839. 32823943 PMC7461582

[ref44] Han T.; Jang Y.; Arvidson A.; D’Souza F.; Wang H. Optical and photophysical properties of platinum benzoporphyrins with C2v and D2h symmetry. J. Porphyrins Phthalocyanines 2022, 26, 458–468. 10.1142/S1088424622500195.

[ref45] Fox S. J. S.; Chen L.; Khan M. A.; Richter-Addo G. B. Nitrosoarene Complexes of Manganese Porphyrins. Inorg. Chem. 1997, 36, 6465–6467. 10.1021/ic970836b.

[ref46] Bichan N. G.; Ovchenkova E. N.; Mozgova V. A.; Kudryakova N. O.; Lomova T. N. Three cobalt(II) porphyrins ligated with pyridyl-containing nanocarbon/gold(III) porphyrin for solar cells: Synthesis and characterization. Polyhedron 2021, 203, 115223 10.1016/j.poly.2021.115223.

[ref47] Xia B.; Gao Y.; Ji F.; Ma F.; Ding L.; Zhou Y. Analysis of Compounds Dissolved in Nonpolar Solvents by Electrospray Ionization on Conductive Nanomaterials. J. Am. Soc. Mass Spectrom. 2018, 29, 573–580. 10.1007/s13361-017-1873-y. 29372550

[ref48] Kadish K. M.; Bottomley L. A.; Beroiz D. Reactions of pyridine with a series of para-substituted tetraphenylporphyrincobalt and -iron complexes. Inorg. Chem. 1978, 17, 1124–1129. 10.1021/ic50183a006.

[ref49] Atoguchi T.; Aramata A.; Kazusaka A.; Enyo M. Cobalt(II)–tetraphenylporphyrin–pyridine complex fixed on a glassy carbon electrode and its prominent catalytic activity for reduction of carbon dioxide. J. Chem. Soc., Chem. Commun. 1991, 156–157. 10.1039/C39910000156.

[ref50] Summers J. S.; Petersen J. L.; Stolzenberg A. M. Comparison of the Structures of the Five-Coordinate Cobalt(II)Pyridine, Five-Coordinate Cobalt(III) Methyl, and Six-Coordinate Cobalt(III) Methyl Pyridine Complexes of Octaethylporphyrin. J. Am. Chem. Soc. 1994, 116, 7189–7195. 10.1021/ja00095a022.

[ref51] Fukuzumi S.; Miyamoto K.; Suenobu T.; Van Caembelcke E.; Kadish K. M. Electron Transfer Mechanism of Organocobalt Porphyrins. Site of Electron Transfer, Migration of Organic Groups, and Cobalt-Carbon Bond Energies in Different Oxidation States. J. Am. Chem. Soc. 1998, 120, 2880–2889. 10.1021/ja973257e.

[ref52] Lebedeva N. S.; Antina E. V.; V’ugin A. I.; Zelenkevich V. Thermodynamics of Formation of Molecular Complexes of Metalloporphyrins with Pyridine in Organic Solvents at 298.15 K. Russ. J. Coord. Chem. 2001, 27, 167–171. 10.1023/A:1009542110838.

[ref53] Manbeck G. F.; Fujita E. A review of iron and cobalt porphyrins, phthalocyanines and related complexes for electrochemical and photochemical reduction of carbon dioxide. J. Porphyrins Phthalocyanines 2015, 19, 45–64. 10.1142/S1088424615300013.

[ref54] Tsutsui M.; Velapoldi R. A.; Hoffman L.; Suzuki K.; Ferrari A. Unusual Metalloporphyrins. III. Induced Oxidation of Cobalt(II) and Iron (II) Porphyrins by Unsaturated Hydrocarbons. J. Am. Chem. Soc. 1969, 91, 3337–3341. 10.1021/ja01040a040.

[ref55] Sugimoto H.; Nobuhiro U.; Masayasu M. Preparation and Physicochemical Properties of Tervalent Cobalt Complexes of Porphyrins. Bull. Chem. Soc. Jpn. 1981, 54, 3425–3432. 10.1246/bcsj.54.3425.

[ref56] Sugimoto H.; Ueda N.; Mori M. Syntheses of Tervalent Iridium Complexes of Octaethylporphyrin; Influence of Axial Ligands on their Ultra-violet–Visible, Infrared, and Nuclear Magnetic Resonance Spectra, and Redox Potentials. J. Chem. Soc., Dalton Trans. 1982, 1611–1616. 10.1039/DT9820001611.

[ref57] Heath D. F.; Mattocks A. R. Preparation of ^14^C-Labelled Dialkylnitrosamines, and an Improved Preparation of *N*-Methyl-*N*-t-Butylamine. J. Chem. Soc. 1961, 4226–4229. 10.1039/JR9610004226.

[ref58] Zolfigol M. A. Efficient and Chemoselective N-Nitrosation of Secondary Amines Under Mild and Heterogeneous Conditions with Sodium Nitrite and Oxalic Acid Two Hydrate. Synth. Commun. 1999, 29, 905–910. 10.1080/00397919908086051.

[ref59] Maguta M. M.; Stenstrøm Y.; Nielsen C. J. Kinetic and Theoretical Study of the Nitrate (NO_3_) Radical Gas Phase Reactions with *N*-Nitrosodimethylamine and *N*-Nitrosodiethylamine. J. Phys. Chem. A 2016, 120, 6970–6977. 10.1021/acs.jpca.6b05440. 27509322

[ref60] Zolfigol M. A.; Ghaemi E.; Madrakian E.; Kiany-Borazjani M. An Efficient Method for *N*-Nitrosation of Secondary Amines Under Mild and Heterogeneous Conditions. Synth. Commun. 2000, 30, 2057–2060. 10.1080/00397910008087255.

[ref61] Hanson L. K.; Gouterman M.; Hanson J. C. Porphyrins. XXIX. The Crystal and Molecular Structure and Luminescence of Bis(dimethylamine)etio(I)porphinatorhodium(III) Chloride Dihydrate. J. Am. Chem. Soc. 1973, 95, 4822–4829. 10.1021/ja00796a010. 4741277

[ref62] Pinto S. M. A.; Lourenco M. A. O.; Calvete M. J. F.; Abreu A. R.; Rosado M. T. S.; Burrows H. D.; Pereira M. M. Synthesis of New Metalloporphyrin Triads: Efficient and Versatile Tripod Optical Sensor for the Detection of Amines. Inorg. Chem. 2011, 50, 7916–7918. 10.1021/ic200727f. 21819057

[ref63] Heier P.; Boscher N. D.; Choquet P.; Heinze K. Dual Application of (Aqua)(Chlorido)(Porphyrinato)Chromium(III) as Hypersensitive Amine-Triggered ON Switch and for Dioxygen Activation. Inorg. Chem. 2014, 53, 11086–11095. 10.1021/ic501644z. 25271996

[ref64] Foreman M. M.; Hirsch R. J.; Weber J. M. Effects of Formate Binding to a Bipyridine-Based Cobalt-4N Complex. J. Phys. Chem. A 2021, 125, 7297–7302. 10.1021/acs.jpca.1c06037. 34396777

[ref65] Seebach D.; Enders D. Umpolung of Amine Reactivity. Nucleophilic α-(Secondary Amino)-alkylation via Metalated Nitrosamines. Angew. Chem., Int. Ed. 1975, 14, 15–32. 10.1002/anie.197500151.

[ref66] Babij N. R.; McCusker E. O.; Whiteker G. T.; Canturk B.; Choy N.; Creemer L. C.; De Amicis C. V.; Hewlett N. M.; Johnson P. L.; Knobelsdorf J. A.; Li F.; Lorsbach B. A.; Nugent B. M.; Ryan S. J.; Smith M. R.; Yang Q. NMR Chemical Shifts of Trace Impurities: Industrially Preferred Solvents Used in Process and Green Chemistry. Org. Process Res. Dev. 2016, 20, 661–667. 10.1021/acs.oprd.5b00417.

[ref67] Gutowski H. S.; Saika A. Dissociation, Chemical Exchange, and the Proton Magnetic Resonance in Some Squeous Electrolytes. J. Chem. Phys. 1953, 21, 1688–1694. 10.1063/1.1698644.

[ref68] Goldberg P. K.; Pundsack T. J.; Splan K. E. Photophysical Investigation of Neutral and Diprotonated Free-Base Bis(Arylethynyl)porphyrins. J. Phys. Chem. A 2011, 115, 10452–10460. 10.1021/jp205309f. 21793565

[ref69] Rudine A. B.; DelFatti B. D.; Wamser C. C. Spectroscopy of Protonated Tetraphenylporphyrins with Amino/Carbomethoxy Substituents: Hyperporphyrin Effects and Evidence for a Monoprotonated Porphyrin. J. Org. Chem. 2013, 78, 6040–6049. 10.1021/jo400742f. 23663204

[ref70] Fang Y.; Bhyrappa P.; Ou Z.; Kadish K. M. Planar and Nonplanar Free-Base Tetraarylporphyrins: β-Pyrrole Substituents and Geometric Effects on Electrochemistry, Spectroelectrochemistry, and Protonation/Deprotonation Reactions in Nonaqueous Media. Chem. – Eur. J. 2014, 20, 524–532. 10.1002/chem.201303141. 24302591

[ref71] Stone A.; Fleischer E. B. The Molecular and Crystal Structure of Porphyrin Diacids. J. Am. Chem. Soc. 1968, 90, 2735–2748. 10.1021/ja01013a001.

[ref72] Chirvony V. S.; Hoek A.; Galievsky V. A.; Sazanovich I. G.; Schaafsma T. J.; Holten D. Comparative Study of the Photophysical Properties of Nonplanar Tetraphenylporphyrin and Octaethylporphyrin Diacids. J. Phys. Chem. B 2000, 104, 9909–9917. 10.1021/jp001631i.

[ref73] Kingsbury C. J.; Flanagan K. J.; Eckhardt H. G.; Kielmann M.; Senge M. O. Weak Interactions and Conformational Changes in Core-Protonated A2- and Ax-Type Porphyrin Dications. Molecules 2020, 25, 3195 10.3390/molecules25143195. 32668713 PMC7397311

[ref74] Eisner U.; Harding J. C. Metalloporphyrins. Part I. Some novel demetallation reactions. J. Chem. Soc. 1964, 4089–4101. 10.1039/JR9640004089.

[ref75] Honda T.; Kojima T.; Fukuzumi S. Control of electron-transfer reduction by protonation of zinc octabutoxyphthalocyanine assisted by intramolecular hydrogen bonding. Chem. Commun. 2011, 47, 7986–7988. 10.1039/C1CC12710A. 21681320

[ref76] Yamamoto E.; Kan-no H.; Tomita N.; Ando D.; Miyazaki T.; Izutsu K. Isolation of *N*-nitrosodimethylamine from drug substances using solid-phase extraction-liquid chromatography–tandem mass spectrometry. J. Pharm. Biomed. Anal. 2022, 210, 114561 10.1016/j.jpba.2021.114561. 34974238

[ref77] Herzog B.; Neier R. Synthesis and characterization of Pi-extended porphyrins as potential precursors for the formation of columnar mesophases: Design principles for columnar mesophases need revision?. Arkivoc 2011, 6, 29–44. 10.3998/ark.5550190.0012.604.

