# Correction to “Does the Red Shift in UV–Vis Spectra Really Provide a Sensing Option for Detection of *N*-Nitrosamines Using Metalloporphyrins?”

**DOI:** 10.1021/acsomega.4c08155

**Published:** 2024-11-20

**Authors:** Marko Trampuž, Mateja Žnidarič, Fabrice Gallou, Zdenko Časar

Upon careful review, we have identified an error in Figure S13 in the originally published article. The ^1^H NMR spectra were incorrectly annotated from top to bottom as **a** to **d**. The correct annotation order, from top to bottom on the right-hand side of the spectra, should be **d** to **a**. The corrected Figure S13 is given below. Consequently, the figure citation in the following sentence on page 1163 of the manuscript: "Furthermore, we evaluated the TPP/NDMA (by supplier B) interaction (Figures 9b and S13b)." should be corrected from "S13b" to "S13c".

**Figure S13:**
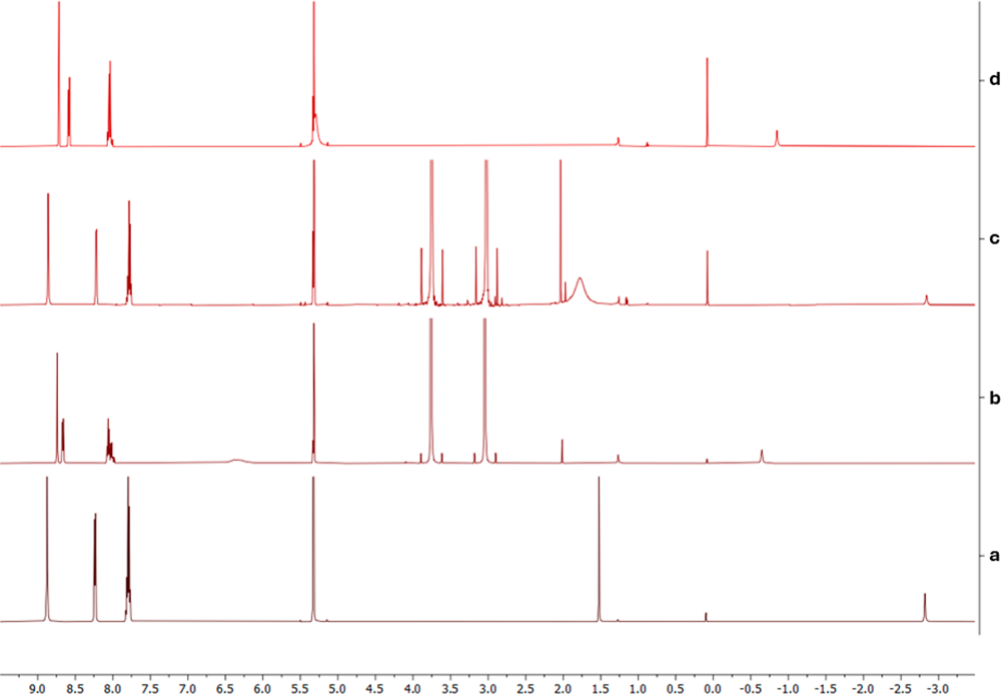
The interaction of various batches of NDMA with TPP as studied by ^1^H NMR spectroscopy in comparison with the interaction of trifluoroacetic acid (TFA) with TPP. The spectra (CD_2_Cl_2_) are as follows: (a) TPP, (b) TPP + 50 eq of NDMA (Supplier A), (c) TPP with 50 eq of NDMA (Supplier B), and (d) TPP + 15 eq of TFA.

