# Supplementary material for: Does the Red Shift in UV–Vis Spectra Really Provide a Sensing Option for Detection of N-Nitrosamines Using Metalloporphyrins?
Source: ACS Omega. 2022 Dec 20;8(1):1154–67. doi: 10.1021/acsomega.2c06615 (PMC9835193; doi:10.1021/acsomega.2c06615)
Supplement: Supplementary file 1 — ao2c06615_si_001.pdf [file ao2c06615_si_001.pdf]

Does the Red Shift in UV-Vis Spectra Really Provide a Sensing Option for Detection of *N*-nitrosamines Using Metalloporphyrins?

Marko Trampuž[a], Mateja Žnidarič[a], Fabrice Gallou[c], and Zdenko Časar[a,b,*]

[a] Lek Pharmaceuticals d.d., Sandoz Development Center Slovenia, SI-1234 Mengeš, Slovenia

[b] University of Ljubljana, Faculty of Pharmacy, Chair of Medicinal Chemistry, Aškerčeva cesta 7, SI-1000, Ljubljana, Slovenia

[c] Chemical and Analytical Development, Novartis Pharma AG, Basel 4056, Switzerland

Contents of Supporting Information:

1. Additional spectroscopic data S-2

Figure S1. UV-Vis spectra of different possibilities for porphyrin F structure S-2

Figure S2. UV-Vis spectra of *meso*-tetra-(4-octoxylphenyl) cobalt porphyrin titrated with 1-50 Eq. of NNN, NNK, NAB & NAT S-3

Figure S3. UV-Vis spectra of *meso*-tetra-(4-octylphenyl) cobalt porphyrin titrated with 1-50 Eq. of NNN & NNK; & 1-10 Eq. of NAB & NATS-3

Figure S4. UV-Vis spectra of metalloporphyrin CY-B titrated with 1-50 Eq. of NNN, NNK, NAB & NAT S-4

Figure S5. ESI-MS spectra of metalloporphyrin CY-B with NNN, NNK, NAB & NAT S-4

Figure S6. ^{1}H NMR spectra of Co(tpp), Co(tpp) with 10 Eq. of NDMA (Supplier A) & Co(tpp) with 10 Eq. of NDMA (Supplier B). S-5

Figure S7. FT-IR spectra of NDMA by supplier A, NDMA by supplier B, Co(TPP)ClO_4, Co(TPP)ClO4 with NDMA by supplier A & Co(TPP)ClO_4 with NDMA by supplier B S-6

Figure S8. UV-VIS spectra of metalloporphyrin CY-B titrated with 1-50 Eq. of *N*-nitroso-2-phenylpyrrolidine (NPP) and *N*-nitrosopiperidine (NPIP), & Porphyrin F titrated with 1-50 Eq. of NPP & NPIP S-6

Figure S9. Structure of nornicotine; Structure of anabasine. UV-VIS spectra of metalloporphyrin CY-B titrated with 1-50 Eq. of pyrrolidine (PYR) & piperidine (PIP), & porphyrin F titrated with 1-50 Eq. of PYR & PIP S-7

Figure S10. UV-VIS spectra of Co(TPP) titrated with 1-50 Eq. of pyridine (Pyr), 1,1-dimethylhydrazine (DMH), trifluoroacetic acid (TFA), & acetic acid (AA) S-7

Figure S11. UV-VIS spectra of Co(TPP)ClO_4 titrated with 1-50 Eq. of pyridine (Pyr), 1,1-dimethylhydrazine (DMH), trifluoroacetic acid (TFA), & acetic acid (AA) S-8

Figure S12. FT-IR spectra of different NDMA batches, provided by Supplier A, Supplier A, neutralized with K_2CO_3, & Supplier B. S-8

Figure S13. The interaction of various batches of NDMA with TPP as studied by ^{1}H NMR spectroscopy in comparison with the interaction of trifluoroacetic acid (TFA) with TPP S-9

Figure S14 UV-Vis spectra of selected acid-labile metalloporphyrins Cd^{2+}(TPP), Er^{3+}(TPP), Eu^{3+}(TPP), Gd^{3+}(TPP), & Pb^{2+}(TPP) before, & after the addition of 40 Eq. of NDMA by Supplier A S-9

Figure S15. General structure of (metallo)phthalocyanines & UV-Vis spectra of Co$(OBu)_8$Pc titrated with 3-50 Eq. of NDMA (Supplier A)S-10

1. **Additional spectroscopic data**

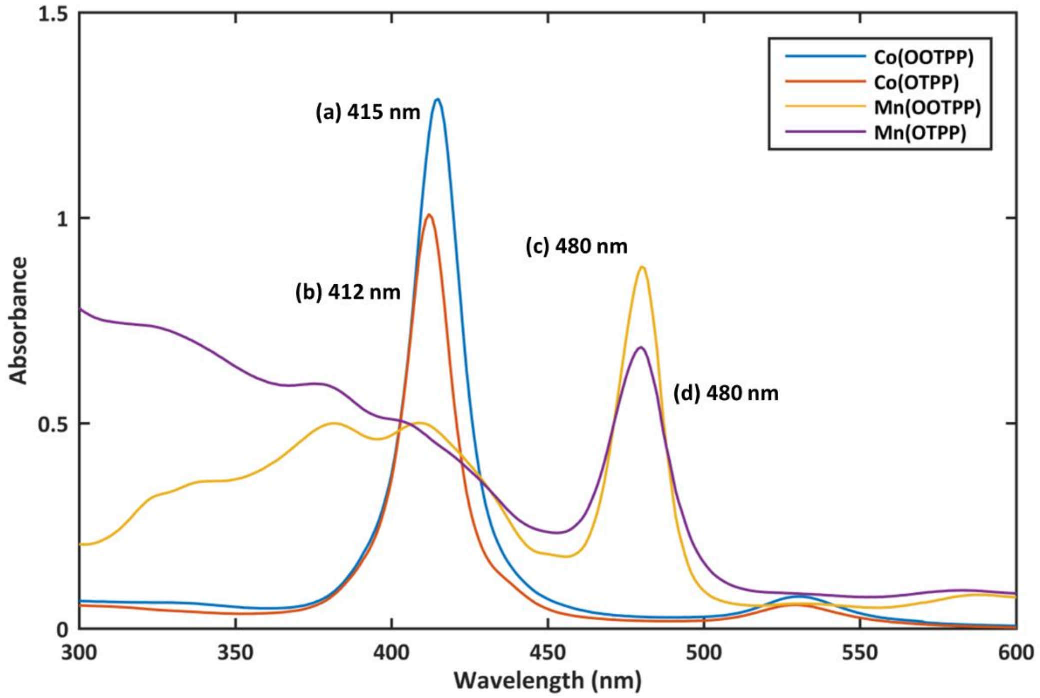

Figure S1: UV-Vis spectra of different possibilities for porphyrin F structure: (a) *meso*-tetra-(4-octoxylphenyl) cobalt porphyrin, (b) *meso*-tetra-(4-octylphenyl) cobalt porphyrin, (c) *meso*-tetra-(4-octoxylphenyl) manganese porphyrin, and (d) *meso*-tetra-(4-octylphenyl) manganese porphyrin. Both Co compounds have Soret band wavelengths in the same region as the published porphyrin F compound (414 nm).

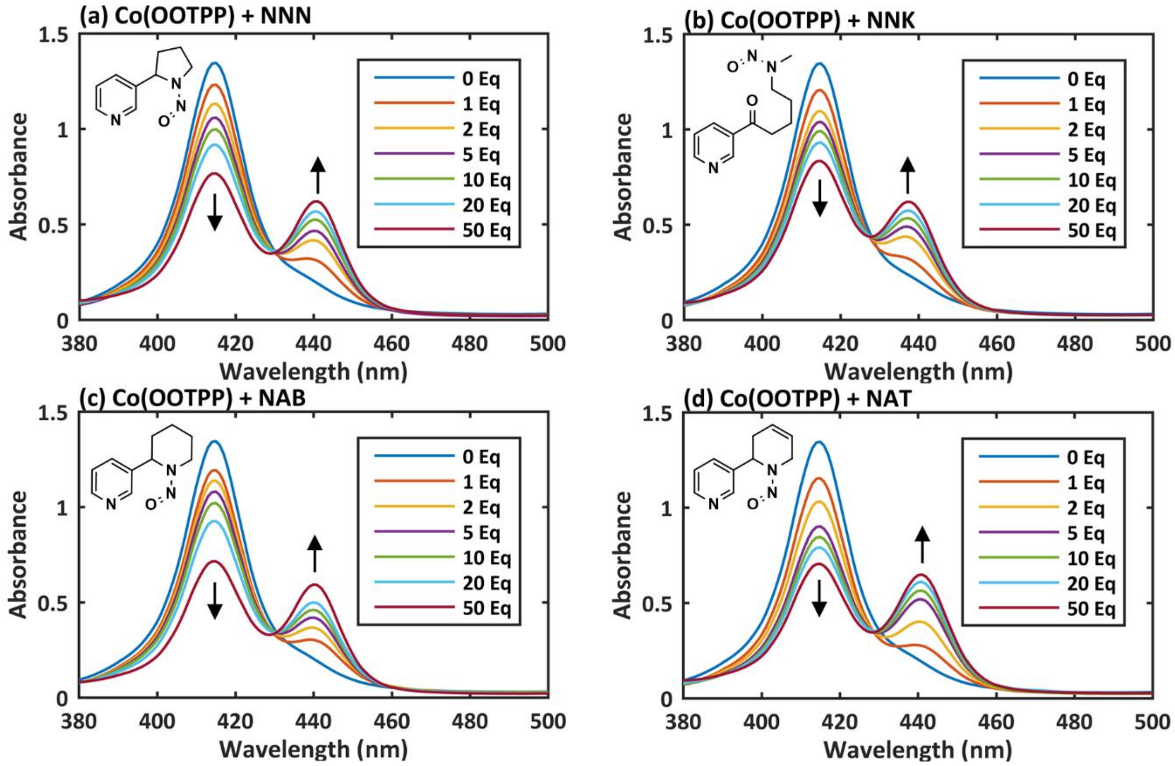

Figure S2: UV-Vis spectra of *meso*-tetra-(4-octoxylphenyl) cobalt porphyrin (first of the two possible porphyrin F structures) titrated with 1-50 Eq. of (a) NNN, (b) NNK, (c) NAB, and (d) NAT.

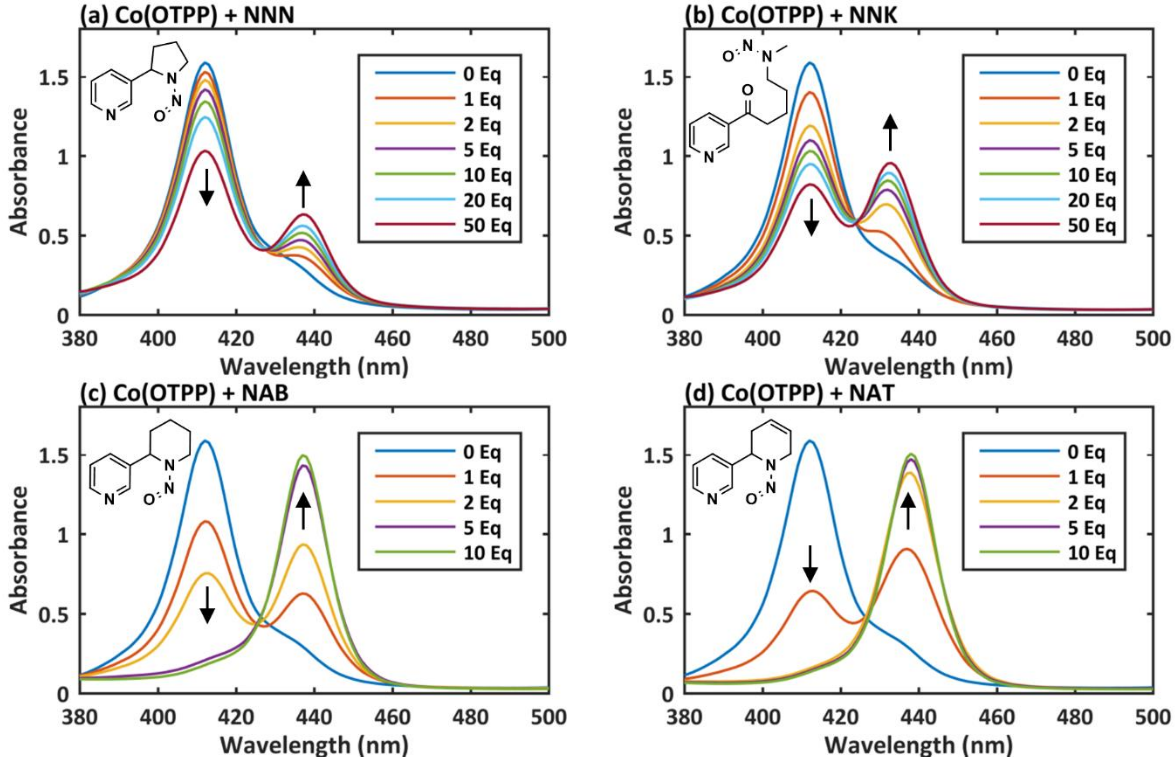

Figure S3: UV-Vis spectra of *meso*-tetra-(4-octylphenyl) cobalt porphyrin (second of the two possible porphyrin F structures) titrated with 1-50 Eq. of (a) NNN and (b) NNK, and 1-10 Eq. of (c) NAB and (d) NAT.

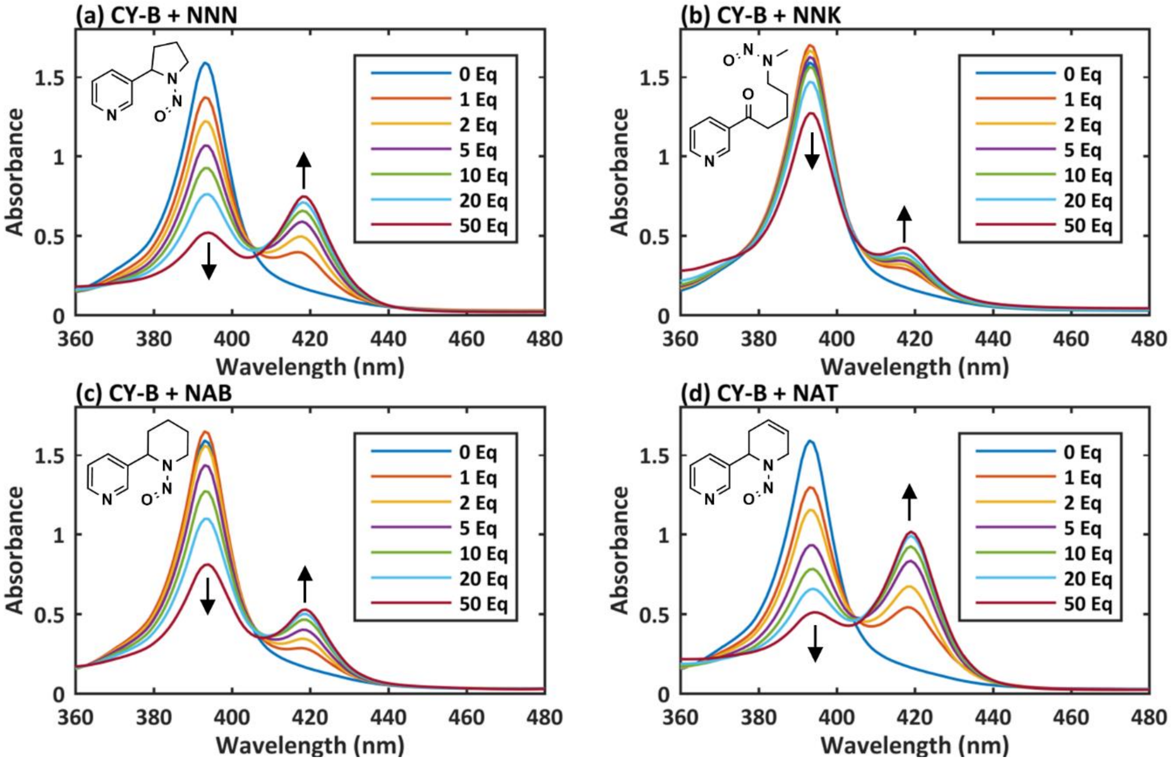

Figure S4: UV-Vis spectra of metalloporphyrin CY-B titrated with 1-50 Eq. of (a) NNN, (b) NNK, (c) NAB, and (d) NAT.

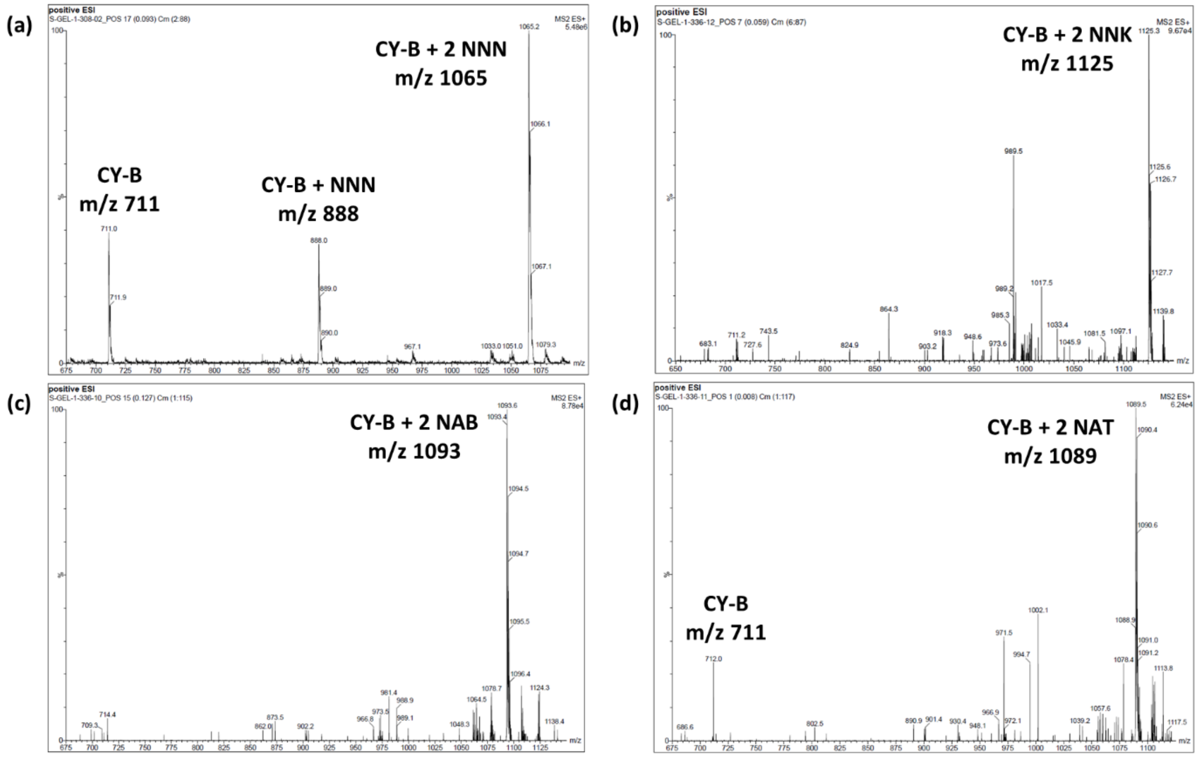

Figure S5: ESI-MS spectra of metalloporphyrin CY-B with (a) NNN, (b) NNK, (c) NAB, and (d) NAT.

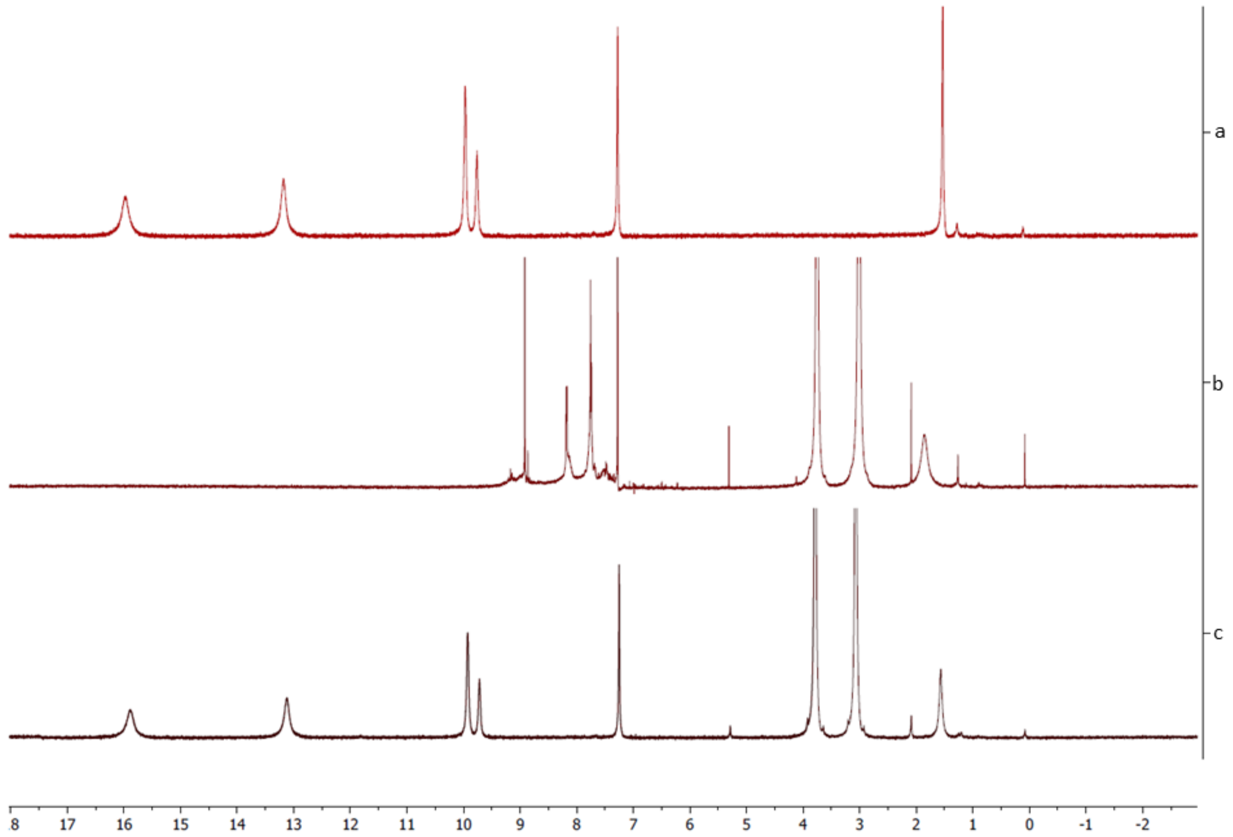

Figure S6: ^{1}H NMR spectra of (a) Co(tpp), (b) Co(tpp) with 10 Eq. of NDMA (Supplier A) and (c) Co(tpp) with 10 Eq. of NDMA (Supplier B).

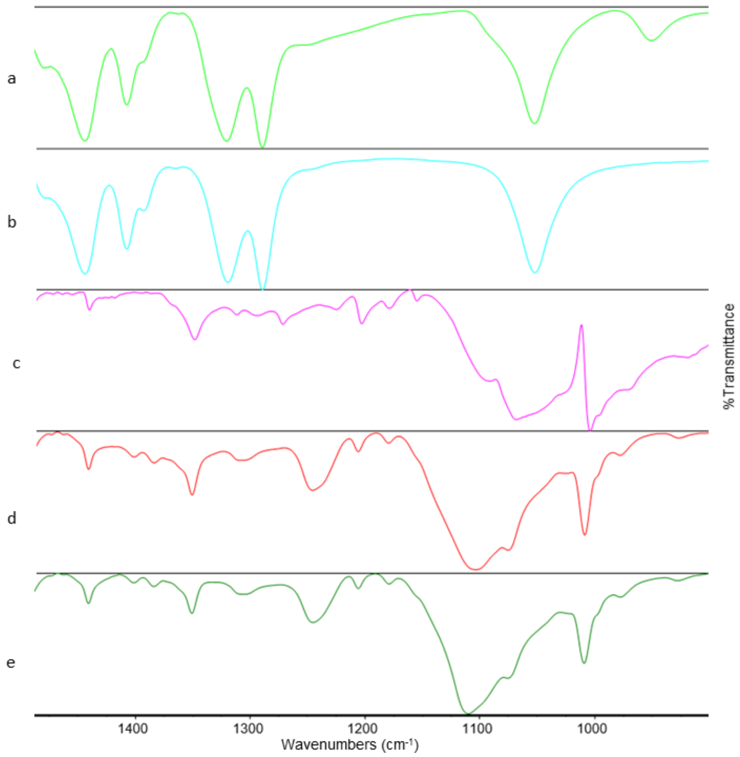

Figure S7: FT-IR spectra of (a) NDMA by supplier A, (b) NDMA by supplier B, (c) Co(TPP)ClO_4, (d) Co(TPP)ClO_4 with NDMA by supplier A and (e) Co(TPP)ClO_4 with NDMA by supplier B.

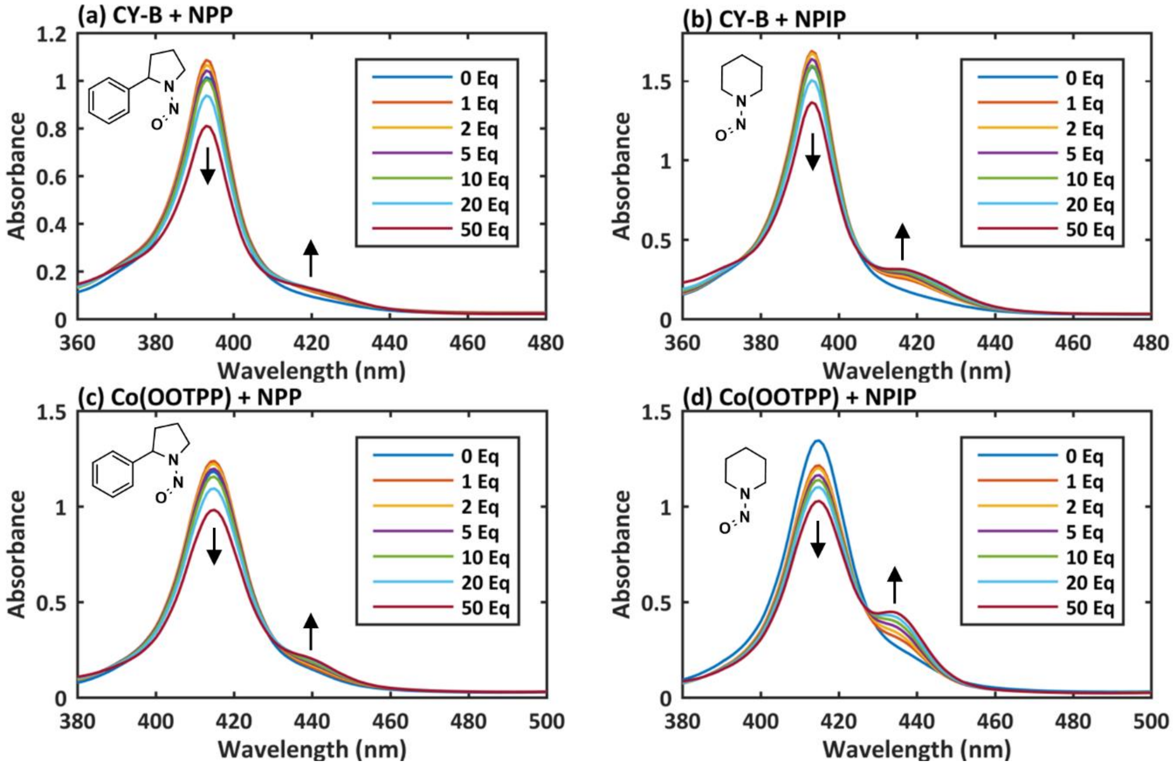

Figure S8: UV-VIS spectra of metalloporphyrin CY-B titrated with 1-50 Eq. of a) *N*-nitroso-2-phenylpyrrolidine (NPP) and b) *N*-nitrosopiperidine (NPIP), and Porphyrin F titrated with 1-50 Eq. of c) NPP and d) NPIP.

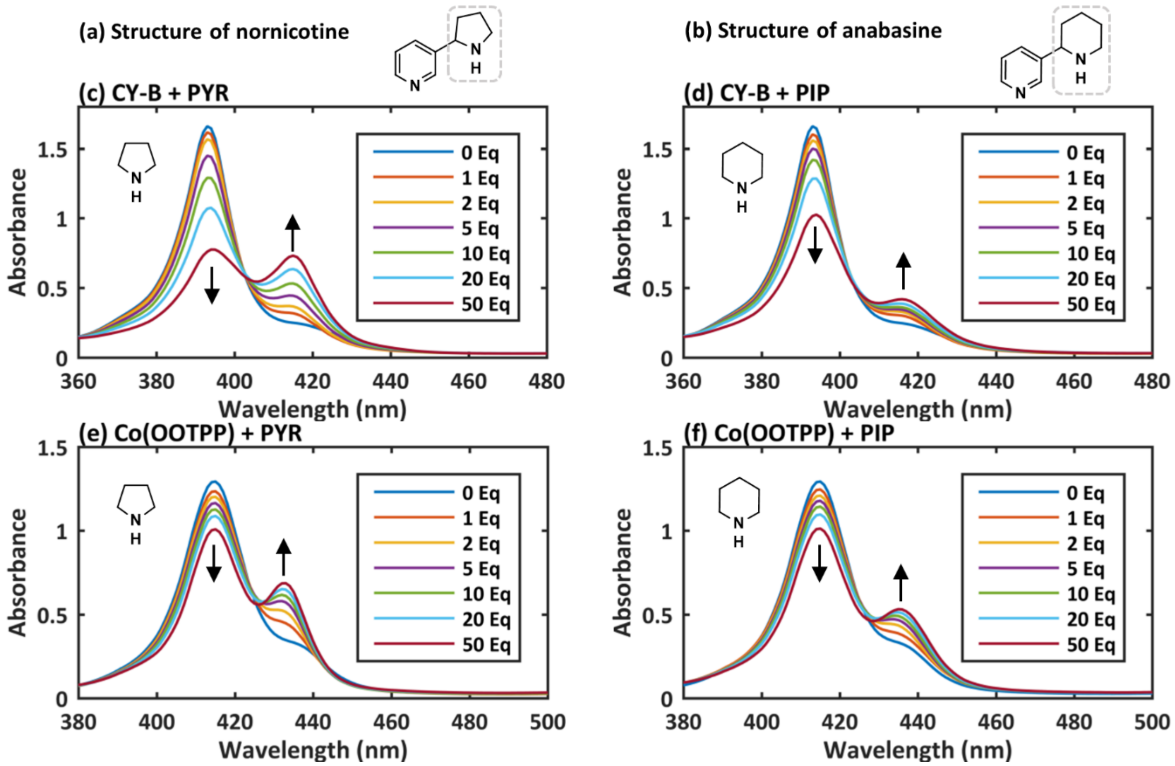

Figure S9: (a) Structure of nornicotine; (b) Structure of anabasine. UV-VIS spectra of metalloporphyrin CY-B titrated with 1-50 Eq. of c) pyrrolidine (PYR) and d) piperidine (PIP), and Porphyrin F titrated with 1-50 Eq. of e) PYR and f) PIP.

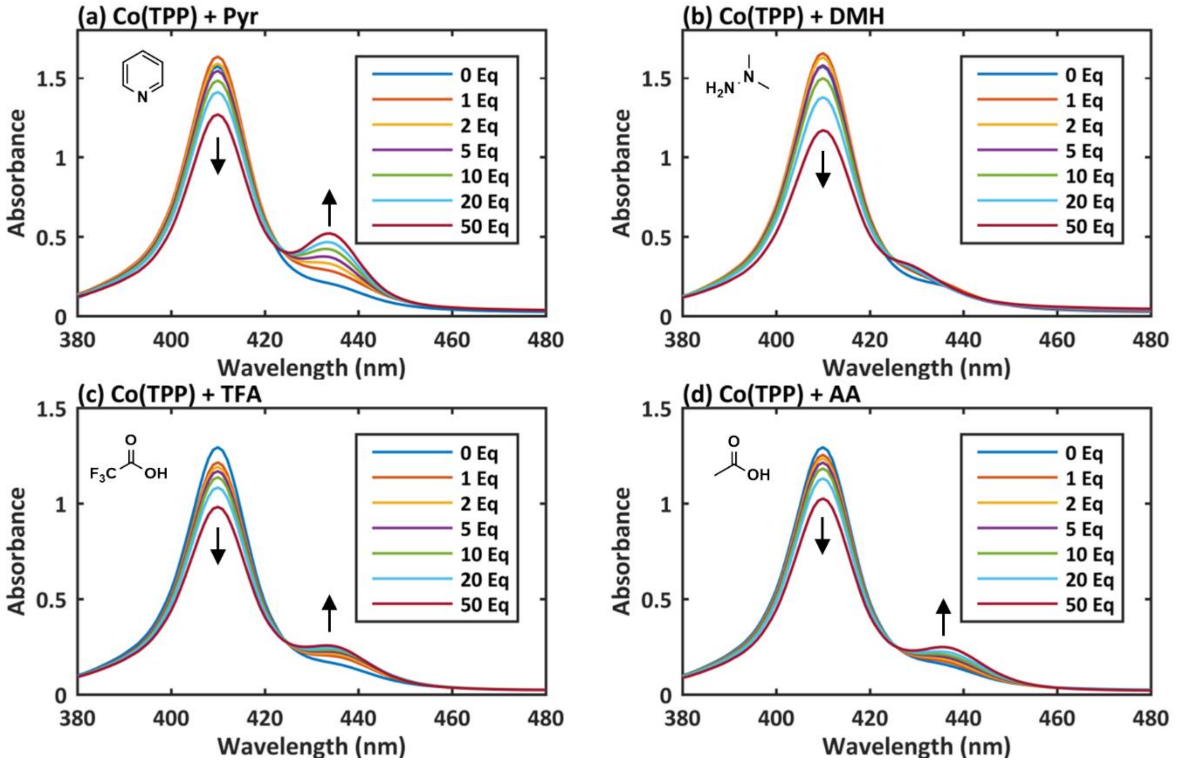

Figure S10: UV-VIS spectra of Co(TPP) titrated with 1-50 Eq. of (a) pyridine (Pyr), (b) 1,1-dimethylhydrazine (DMH), (c) trifluoroacetic acid (TFA), and (d) acetic acid (AA).

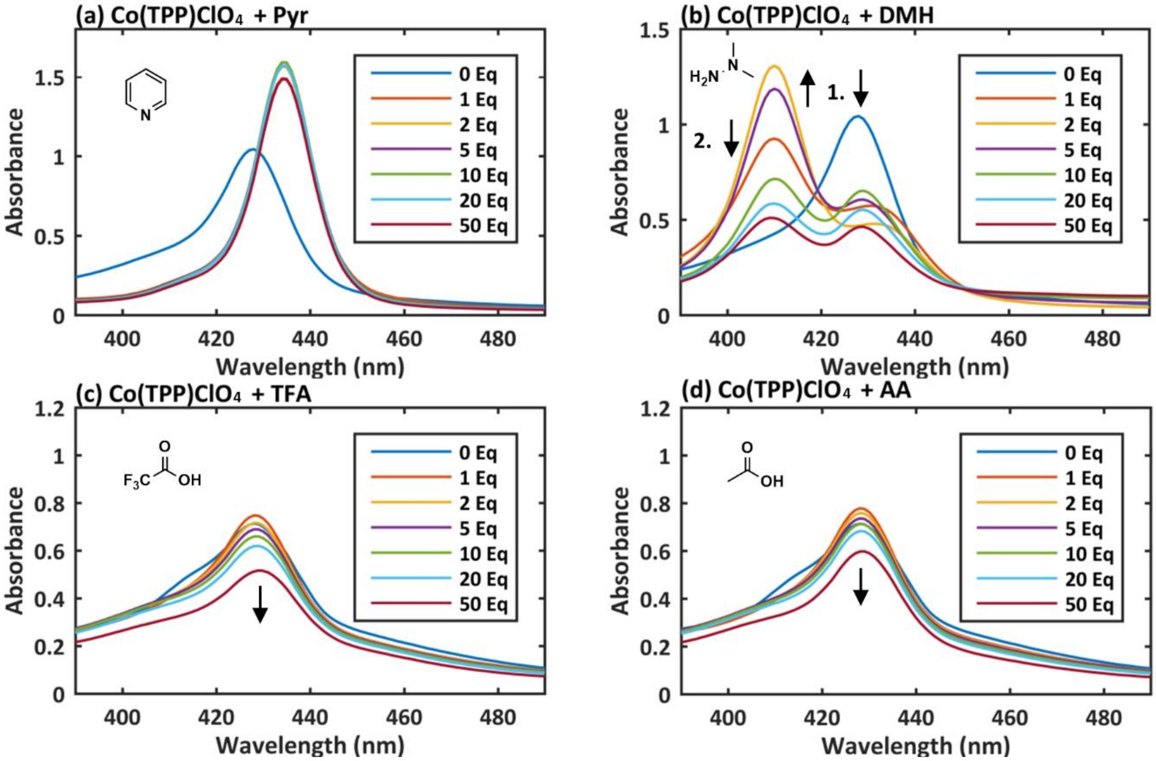

Figure S11: UV-VIS spectra of Co(TPP)ClO$_4$ titrated with 1-50 Eq. of (a) pyridine (Pyr), (b) 1,1-dimethylhydrazine (DMH), (c) trifluoroacetic acid (TFA), and (d) acetic acid (AA).

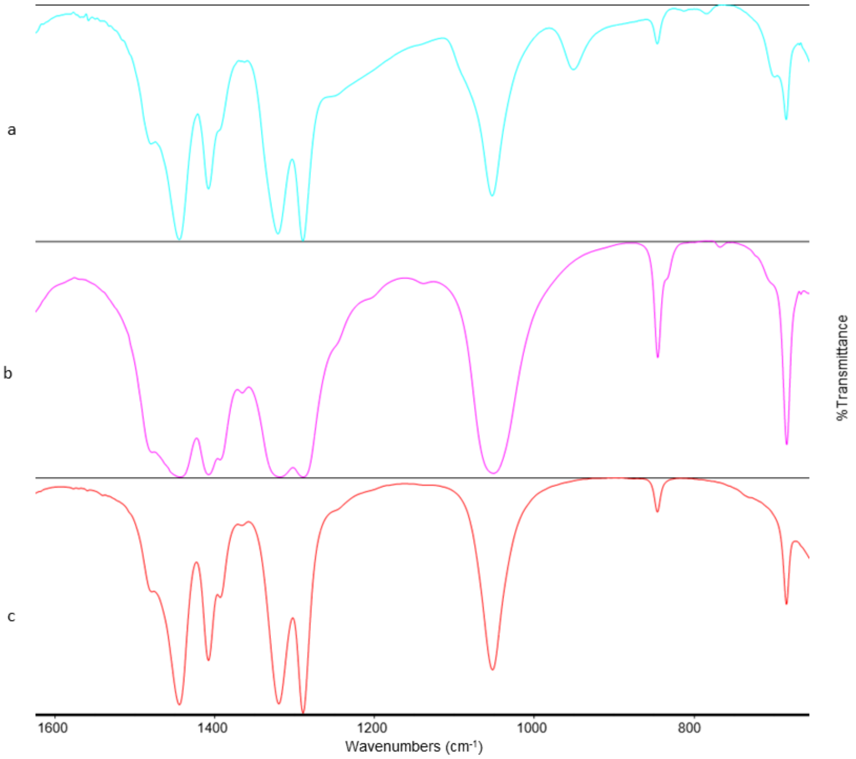

Figure S12: FT-IR spectra of different NDMA batches, provided by (a) Supplier A, (b) Supplier A, neutralized with K$_2$CO$_3$, and (c) Supplier B.

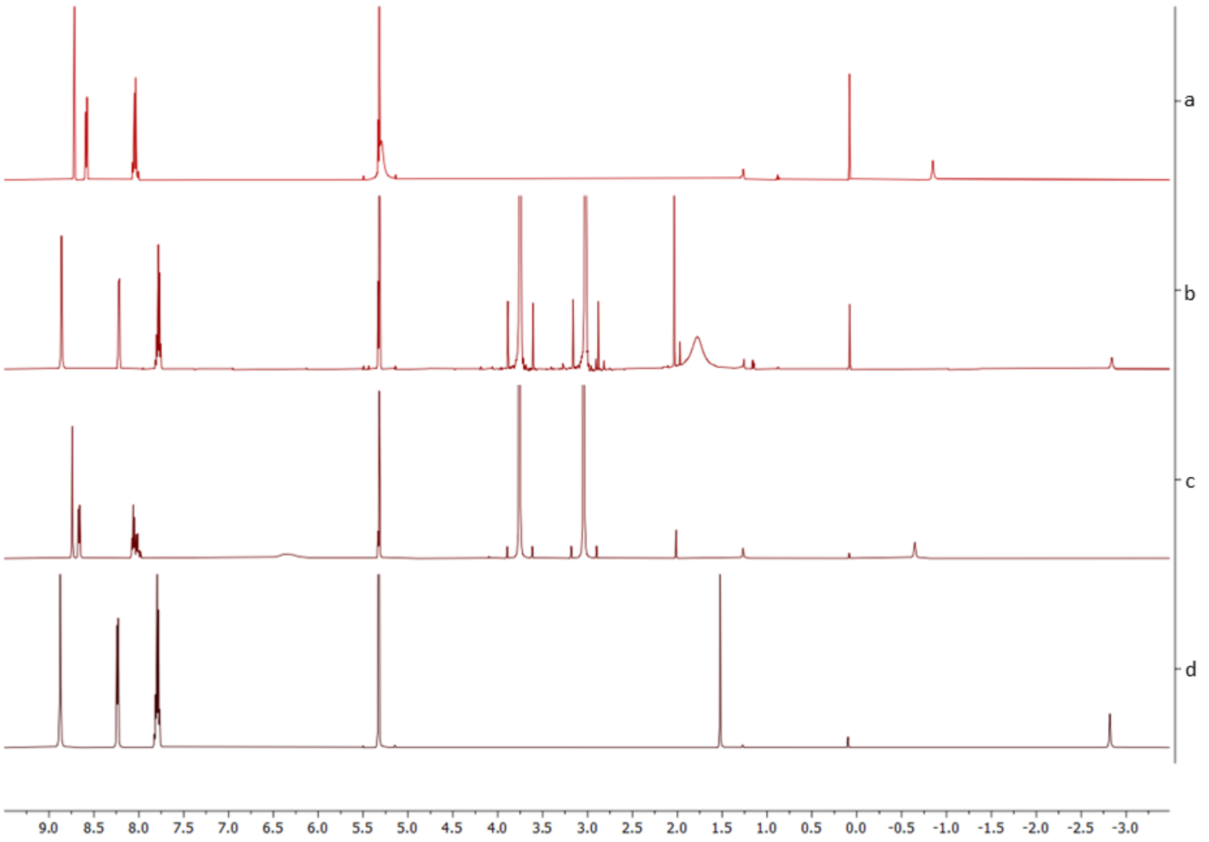

Figure S13: The interaction of various batches of NDMA with TPP as studied by ^{1}H NMR spectroscopy in comparison with the interaction of trifluoroacetic acid (TFA) with TPP. The spectra (CD_2Cl_2) are as follows: (a) TPP, (b) TPP + 50 Eq. of NDMA (Supplier A), (c) TPP with 50 Eq. of NDMA (Supplier B), and (d) TPP + 15 Eq. of TFA.

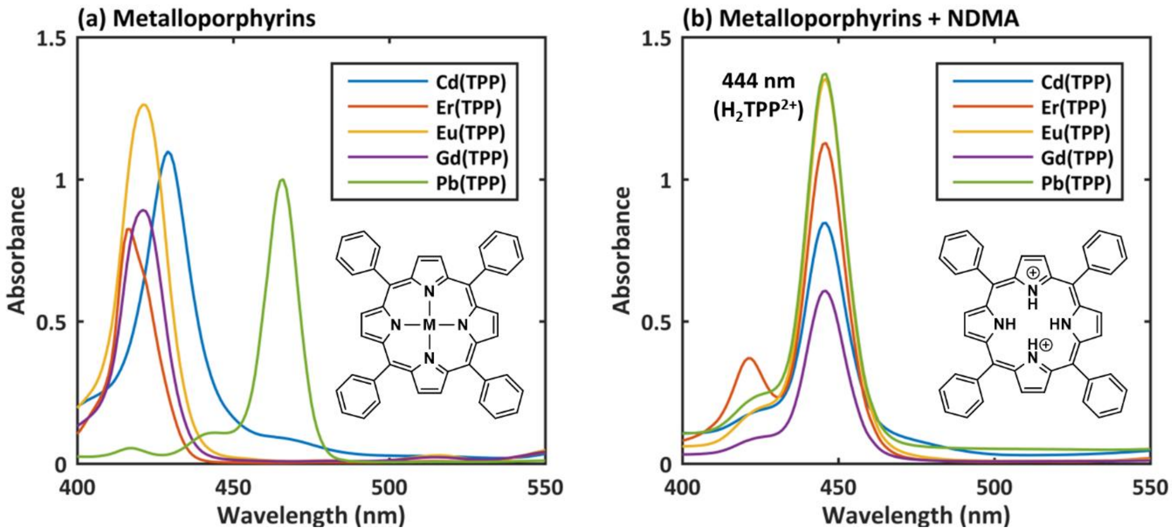

Figure S14: UV-Vis spectra of selected acid-labile metalloporphyrins Cd^{2+}(TPP), Er^{3+}(TPP), Eu^{3+}(TPP), Gd^{3+}(TPP), and Pb^{2+}(TPP) (a) before, and (b) after the addition of 40 Eq. of NDMA by Supplier A.

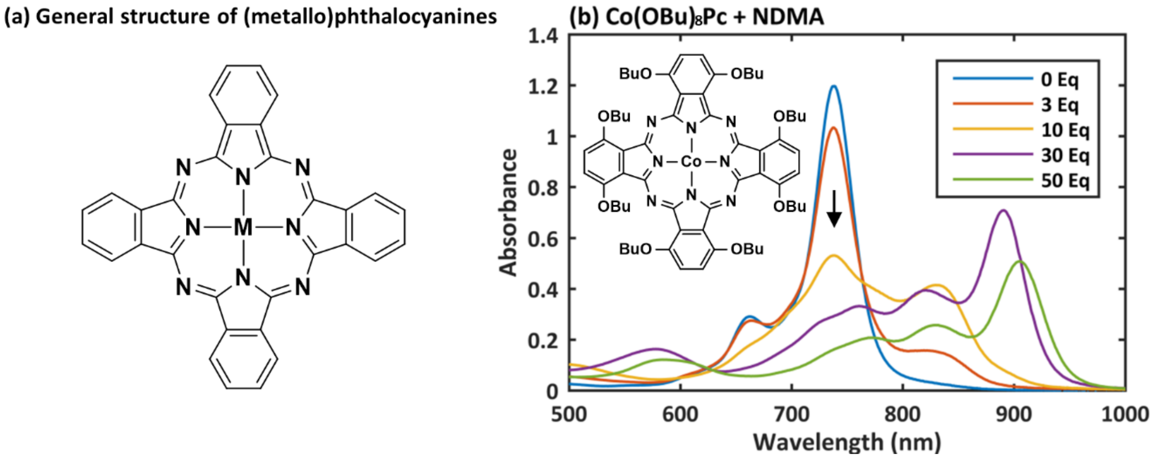

Figure S15: (a) General structure of (metallo)phthalocyanines and (b) UV-Vis spectra of $Co(OBu)_8Pc$ titrated with 3-50 Eq. of NDMA (Supplier A).
